# Supplementary material for: A Literature-Derived Knowledge Graph Augments the Interpretation of Single Cell RNA-seq Datasets
Source: Genes (Basel). 2021 Jun 10;12(6):898. doi: 10.3390/genes12060898 (PMC8229796; doi:10.3390/genes12060898)
Supplement: Supplementary file 1 [file genes-12-00898-s001.zip › Single Cell Literature Annotation Manuscript Revision1 SUPPLEMENTAL COPYEDIT VERSION CLEAN.pdf]

SUPPLEMENTAL INFORMATION

A Literature-Derived Knowledge Graph Augments the Interpretation of Single Cell RNA-seq Datasets

Deeksha Doddahonnaiah*[1], Patrick J. Lenehan*[1], Travis K. Hughes[1], David Zemmour[1], Enrique Garcia-Rivera[1], A.J. Venkatakrishnan[1], Ramakrishna Chilaka[2], Apoorv Khare[2], Akhil Kasaraneni[2], Abhinav Garg[2], Akash Anand[2], Rakesh Barve[2], Viswanathan Thiagarajan[2], Venky Soundararajan[#1,2]

[1] nference, One Main Street, Cambridge, MA 02142, USA
[2] nference Labs, Bengaluru, India
* Contributed equally
[#] Corresponding author

Supplemental Methods

Cell type annotation algorithm

To perform automated annotation of cell types (clusters) identified from scRNA-seq datasets using our literature derived knowledge graph, we performed the following steps: (1) identify the top N cluster defining genes (CDGs), (2) compute local scores between these CDGs and all candidate cell types, (3) compute local score vector norms for each candidate cell type, and (4) rank candidate cell types for annotation plausibility based on their vector norms. Each of these steps are described in detail below, and an example workflow for annotating a single cluster is illustrated schematically in **Figures 2-3**. The studies which were used to tune the cell type annotation algorithm are listed in **Table 1**, and the studies which were used to test it are listed in **Table 2**.

1. Identify the top N cluster defining genes (CDGs)

To compute cluster defining genes for a given cluster to annotate (C_A) from study *S*, we compared the mean expression of all 5,113 eligible genes in C_A to their mean expression in a reference set (*R*) of single cells. Specifically, we calculated the fold change (FC) and $\log_2$FC of mean expression for each gene, where FC = (Mean CP10K in C_A + 1) / (Mean CP10K in *R* + 1) and $\log_2$FC = $\log_2$(FC).

These results were sorted in descending order and stored as two vectors: a 5,113-dimensional fold change vector *F* ($[f_1, f_2, \ldots, f_{5113}]$) and a 5,113-dimensional $\log_2$FC vector *G* ($[g_1, g_2, \ldots, g_{5113}]$).

These vectors *F* and *G* were scaled to range from 0 to 1 as follows:

- Scaled $f_i = w_i = (f_i - F_{min}) / (F_{max} - F_{min})$
- Scaled $g_i = x_i = (g_i - G_{min}) / (G_{max} - G_{min})$

The top N genes were then selected as CDGs, leading to the creation of two vectors for use in subsequent analyses: a N-dimensional scaled FC vector *W* ($[w_1, w_2, \ldots, w_N]$) and a N-dimensional scaled $\log_2$FC vector *X* ($[x_1, x_2, \ldots, x_N]$).

2. Store absolute and scaled GCAs between these CDGs and all cell types

Absolute and scaled GCAs between the N selected CDGs for C_A and all 556 candidate cell types were extracted from the GCA matrix (described above and given in **Files S5-S6**). This resulted in the generation of 556 N-dimensional vectors of absolute GCAs (one vector per candidate cell type) and 556 N-dimensional vectors of scaled GCAs (one vector per candidate cell type), represented for each candidate cell type as follows:

- Absolute GCA Vector *Y*: $[y_1, y_2, \ldots, y_N]$

- Scaled GCA Vector Z: $[z_1, z_2, \dots, z_N]$

3. Compute GCA vector norms for each cell type

For a given candidate cell type C_C, we started with the N-dimensional vectors defined above, where each dimension corresponds to one of the N top CDGs: scaled FC (W), scaled $\log_2$FC (X), absolute GCAs (Y), scaled GCAs (Z). We then used these vectors to compute various scores quantifying the level of literature evidence connecting C_C to the set of CDGs. The computed scores included variations of L0 and L2 norms as follows:

1. Modified L0 $\text{norm}_{\text{Absolute GCAs}}$ = number of elements in Y greater than or equal to 3
2. L2 $\text{norm}_{\text{Absolute GCAs}} = \text{sqrt}(y_1^2 + y_2^2 + \dots + y_N^2)$
3. FC Weighted L2 $\text{norm}_{\text{Absolute GCAs}} = \text{sqrt}(w_1*y_1^2 + w_2*y_2^2 + \dots + w_N*y_N^2)$
4. Log_2FC Weighted L2 $\text{norm}_{\text{Absolute GCAs}} = \text{sqrt}(x_1*y_1^2 + x_2*y_2^2 + \dots + x_N*y_N^2)$
5. L2 $\text{norm}_{\text{Scaled GCAs}} = \text{sqrt}(z_1^2 + z_2^2 + \dots + z_N^2)$
6. FC Weighted L2 $\text{norm}_{\text{Scaled GCAs}} = \text{sqrt}(w_1*z_1^2 + w_2*z_2^2 + \dots + w_N*z_N^2)$
7. Log_2FC Weighted L2 $\text{norm}_{\text{Scaled GCAs}} = \text{sqrt}(x_1*z_1^2 + x_2*z_2^2 + \dots + x_N*z_N^2)$

Thus, for each candidate cell type C_C, we calculated seven literature-based metrics. These metrics were computed for all 556 candidate cell types, yielding a matrix of 556 candidate cell types by 7 metrics.

4. Rank candidate cell types for annotation plausibility based on their vector norms

This 556 by 7 matrix was then leveraged to predict the most likely cellular identity of the cluster C_A. Specifically, we tested the utility of each individual metric and a combination of the L0 and L2 metrics ("composite ranks") in predicting the correct cellular identity.

When using individual metrics, cell type predictions were ranked by simply sorting the corresponding score in descending order (i.e., the prediction with the highest score was assigned a rank of 1). In the case of ties (i.e., predictions with the same score), all tied predictions were assigned the maximum (worst) possible rank; for example, if 10 predictions were tied for the highest score, then all of them were assigned a rank of 10.

To derive composite ranks, we first determined the mean and minimum of the modified L0 rank and a given L2 rank (e.g. L2 $\text{norm}_{\text{Absolute GCAs}}$) for each cell type prediction. That is, each version of the L2 norm (n = 6) was used to generate a separate composite rank. Predictions were then ranked

by sorting with the following priority order: mean rank (descending), minimum rank (ascending), and modified L0 rank (ascending). Ties were again addressed by assigning the maximum (worst) rank to all tied predictions, as described above for the handling of individual metrics.

Hyperparameter tuning of cell type annotation algorithms

There were five adjustable parameters in our cell type annotation algorithm, which were each tested for their impact on algorithm performance as follows (see **Figure 3**):

1. *Reference set (R) of single cells used to identify the top CDGs for cluster C_A*. We tested three options for this parameter: "within study", "within tissue", and "pan-study." For the "within study" reference, the mean expression of each gene in C_A was compared to its mean expression in all other cells from the same study. For the "within tissue" reference, the mean expression of each gene in C_A was compared to its mean expression in all other cells from any studies which were derived from the same tissue as C_A. For the "pan-study" reference, the mean expression of each gene in C_A was compared to its mean expression in all other cells from all other processed studies (approximately 2.5 million cells; see **File S8**). We tested these options because each has its own advantages and disadvantages. While "within study" comparisons are most commonly performed by investigators when annotating scRNA-seq datasets and are less prone to technical artifacts, selection of cell types prior to sequencing (e.g., by fluorescence activated cell sorting) can lead to the dropout of important cell type defining genes from a CDG list in this analysis workflow. For example, in a scRNA-seq study of sorted $CD8^+$ T cells, important cell type markers (e.g., CD3E, CD8A, CD8B) will be ubiquitously expressed and inherently will not be identified as CDGs for each identified subcluster. On the other hand, the "pan study" and "within tissue" comparisons are more prone to technical artifacts (e.g., batch effects, differences in sequencing depth and sample viability between studies) but are better able to preserve cell type markers in examples like the one described above. It is important to note that the pan-study comparison is also more likely to be adversely impacted by tissue contaminants (e.g., extracellular RNA) than within study or within tissue comparisons. For example, highly expressed transcripts from abundant parenchymal cells (e.g., albumin in the hepatocytes) are often detected in other non-parenchymal cells from the same tissue. When performing a pan-study comparison, this contamination of highly tissue specific transcripts could lead to the incorrect identification of these genes as markers for even the non-parenchymal cell types. However, if the cluster is compared to only other cells from the same study or tissue (which presumably have similar levels of contaminant gene expression), this issue can be avoided.

2. *Number of CDGs used to compute GCA vector norms.* We tested five options for this parameter: 1, 3, 5, 10, and 20. This range of values was selected to mirror the typical manual workflows utilized by investigators annotating their own datasets. In some cases, a single obvious CDG is enough to declare a cellular identity, while in other cases it is necessary to consider the combination of several genes among the top 10 to 20 CDGs.

3. *Weighting metric used in calculating GCA L2 vector norms.* We tested three options for this parameter: no weighting, FC, and log_2FC. The reason for testing this parameter is that it may be reasonable to assign more value to genes that are more strongly overexpressed in cluster C_A when attempting to annotate it. This hyperparameter tuning is also captured in the previous section "Compute local score vector norms for each cell type."

4. *GCA version used to calculate L2 vector norms.* We tested two options for this parameter: absolute and scaled. The scaling of GCAs was described previously, and the raw and scaled GCAs are provided in **Files S5-S6**. This hyperparameter tuning is also captured in the previous section "Compute local score vector norms for each cell type."

5. *Metric used to rank cell type predictions.* We tested three options for this parameter: modified L0 norm rank, L2 norm rank, and composite rank. The derivation of these ranks is described in the previous section "Rank plausible cell type annotations based on their vector norms."

In total, we tested 195 combinations of parameters. Note that this is fewer than the total number of "possible" parameter combinations (3 x 5 x 3 x 2 x 3 = 270) because the weighting metric and GCA version used (absolute vs. scaled) in calculating L2 vector norms were irrelevant for all parameter combinations in which the modified L0 norm rank was used as the metric to rank cell type predictions.

Supplemental Results

Detailed review of annotation results for tuning studies

The predicted annotations using our optimized algorithm parameters are shown for selected tuning studies from retina, blood, and pancreas [1–5] in **Figure S5**. All retinal cell types except for B cells were correctly classified, including retinal pigment epithelial cells, melanocytes, and Schwann cells along with tissue resident immune and stromal cells (**Figures S5A-B**). B cells were incorrectly classified as dendritic cells, which may reflect their shared status as professional antigen presenting cells. That said, the prediction with the second highest rank was indeed B cells (**Figure S5B**).

In the blood, the labeling of monocytes proved difficult, as both $CD14^{+}$ and $CD16^{+}$ monocytes were misclassified as macrophages (**Figures S5C-D**). This likely reflects the close developmental and transcriptional relationships between monocytes and macrophages, as monocytes can differentiate into macrophages upon migration from circulation into tissues [6]. The misclassification of cytotoxic T cells as NK cells is also understandable, given their shared expression of cytolytic effector molecules and the fact that these cells often cluster together in scRNA-seq analyses due to their transcriptional similarity (**Figures S5C-D**).

In the pancreas, this algorithm correctly annotated acinar cells, schwann cells, endothelial cells, macrophages, and mast cells. Ductal cells were correctly classified as epithelial cells, while the specific annotation of ductal cells was ranked fourth. Alpha, beta, delta, epsilon, and gamma cells were all correctly classified as endocrine cells, with their specific subtypes ranked shortly after this broader categorization (**Figures S5E-F**). The classification of stellate cells (both quiescent and activated) as fibroblasts was deemed technically incorrect, although stellate cells are indeed known to display myofibroblast-like properties [7]. That said, stellate cells were the second ranked predictions for each of these two clusters (**Figures S5E-F**).

The predicted annotations for all other tuning studies are shown in **Figures S6-S9**. It was interesting to note that certain studies were more accurately labeled with parameter settings that diverged from the overall optimized settings. For example, in one study of the retina which contained a large population of rod photoreceptors, only three of nine clusters were accurately labeled while several other cell types (e.g. amacrine cells, endothelial cells, and muller glia) were incorrectly classified as rods with the optimized settings (**Figure S9D**). Amacrine cells were particularly problematic, with the correct label receiving a rank of 19. This suggests that many cells were contaminated with rod-specific transcripts at a high enough abundance that they dominated the CDG list when compared to all other cells in our reference set. However, this artifact was substantially mitigated by considering only the top 10 CDGs calculated using the "within-study" method and ranking predictions by the composite metric. With these settings, amacrine cells, endothelial cells, and muller glia were all annotated correctly.

Supplemental Figures

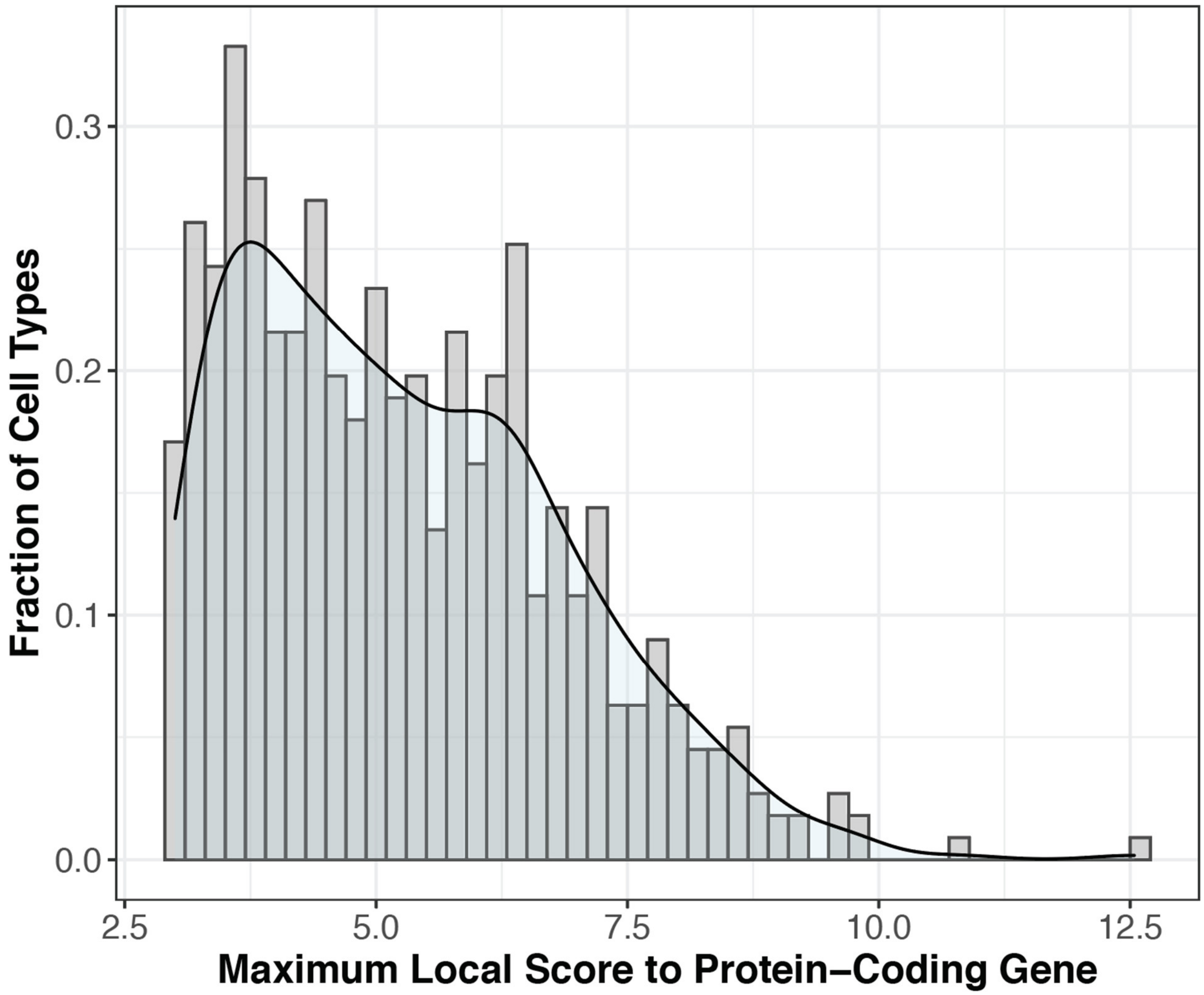

Figure S1. Distribution of maximum GCAs for all 556 candidate cell types. For each candidate cell type (n = 556), the maximum GCA (local score between any human protein-coding gene and the given cell type) was extracted from **File S5**. This distribution shows that the maximum GCA varies substantially by cell type (range 3.00 - 12.54), and so we also scaled these values to range from 0 to 1 for each cell type (see **File S6**). Both absolute and scaled GCAs were tested for their utility in cluster annotation.

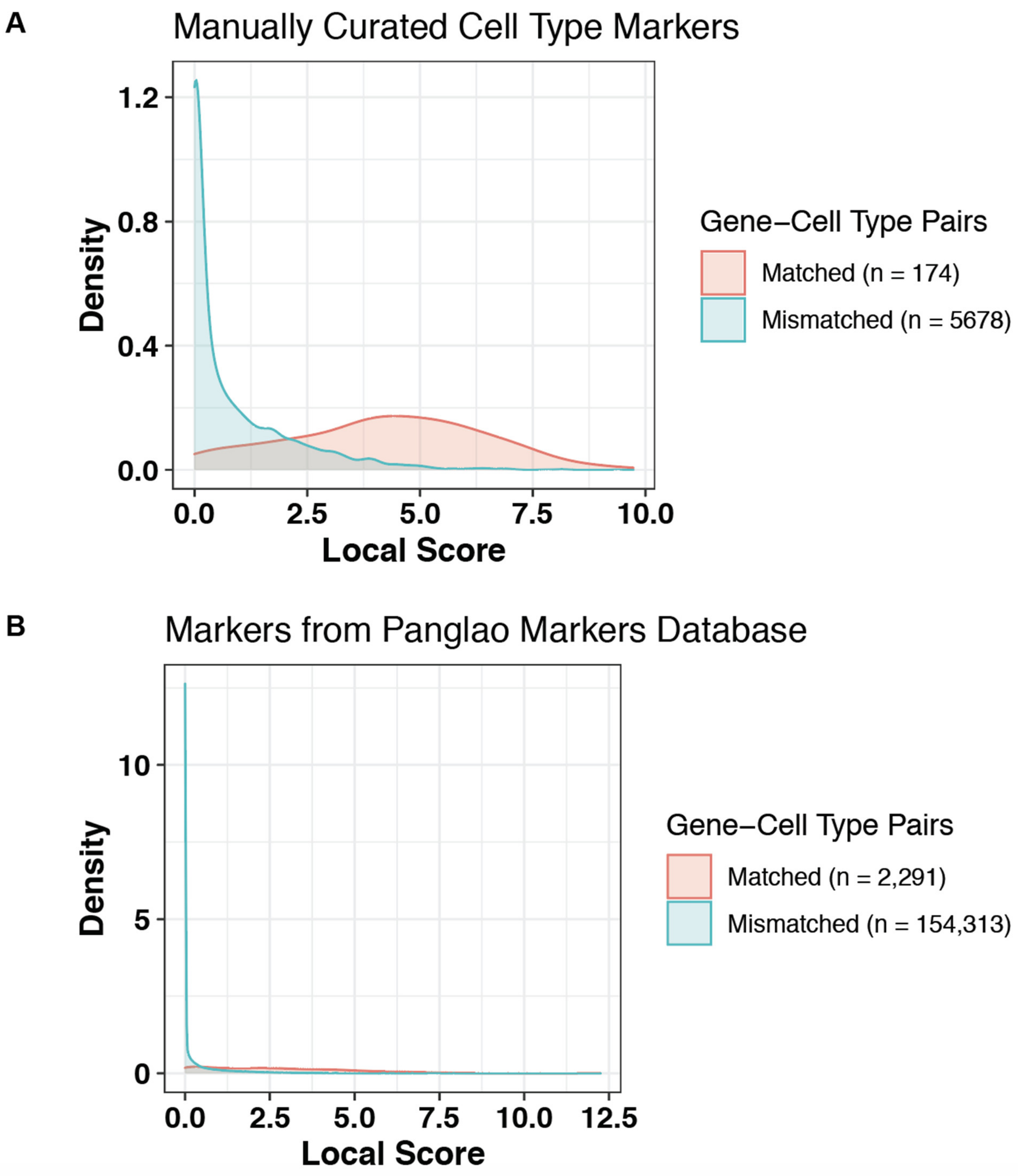

Figure S2. Distribution of gene-cell type local scores (GCAs) between matched and mismatched pairs. A "matched" pair corresponds to (A) a gene which was used to define a cell type in prior scRNA-seq analyses and its corresponding cell type, or (B) a gene which is documented as a canonical human cell type marker in the Panglao database and its corresponding cell type [8]. In each case, after obtaining the set of matched pairs, all other possible gene-cell type combinations (i.e., all other pairwise combinations of these genes and cell types) were considered

“mismatched” pairs. The distributions here show that these local scores do not follow a normal distribution, and so we used nonparametric tests to assess the difference between them.

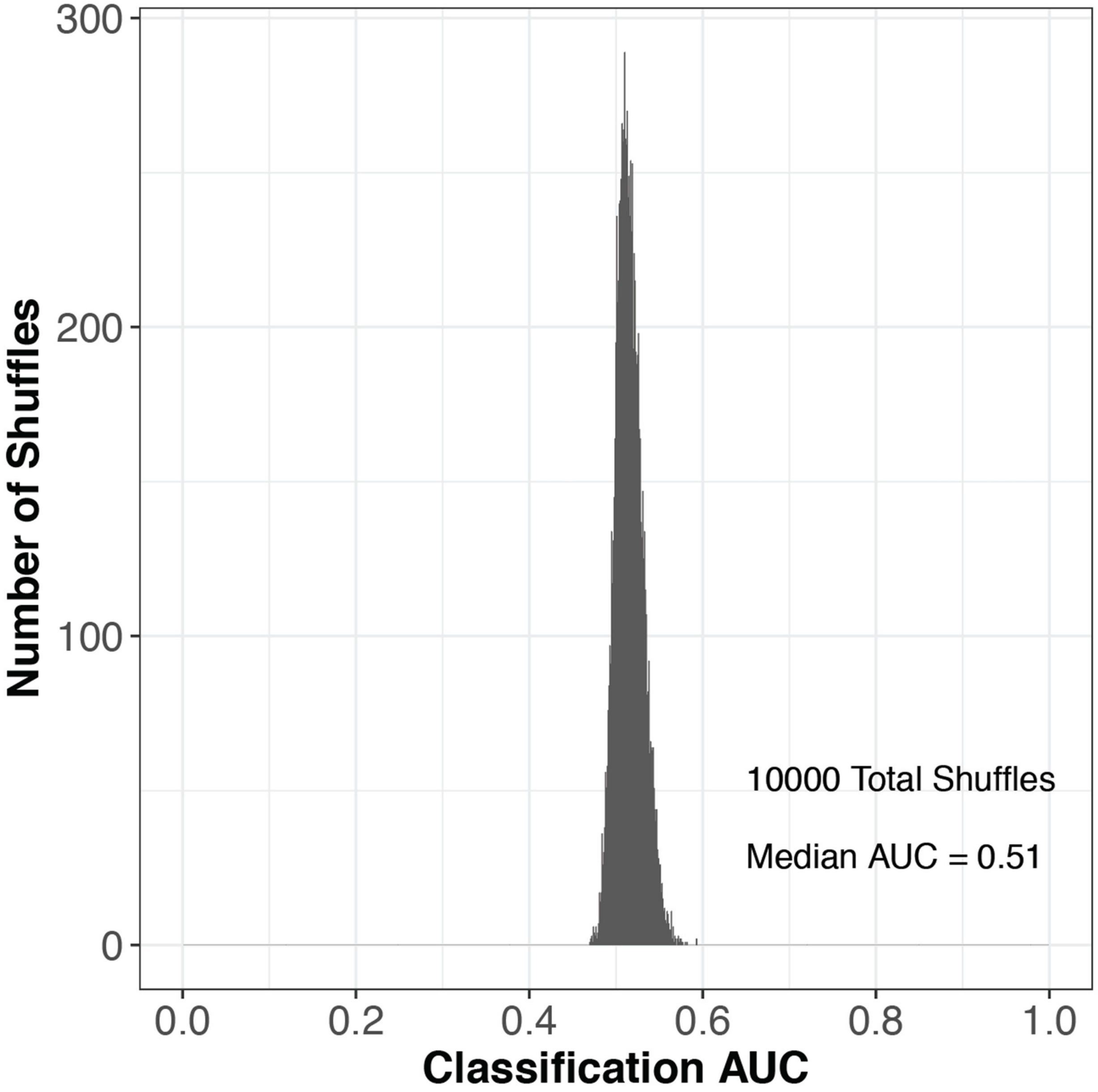

Figure S3. Distribution of AUC values from ROC analysis of GCA-based classification of manually curated gene-cell type pairs with randomly shuffled matched and mismatched assignments. A set of matched gene cell-type pairs was manually curated, and the complement pairs were designated as mismatched. After performing an ROC analysis to determine the classification power of GCAs in discriminating matched from mismatched gene-cell type pairs, we performed 10,000 iterations of this ROC analysis with random shuffling of the matched and mismatched labels. This histogram shows the distribution of the 10,000 AUC values obtained from these analyses.

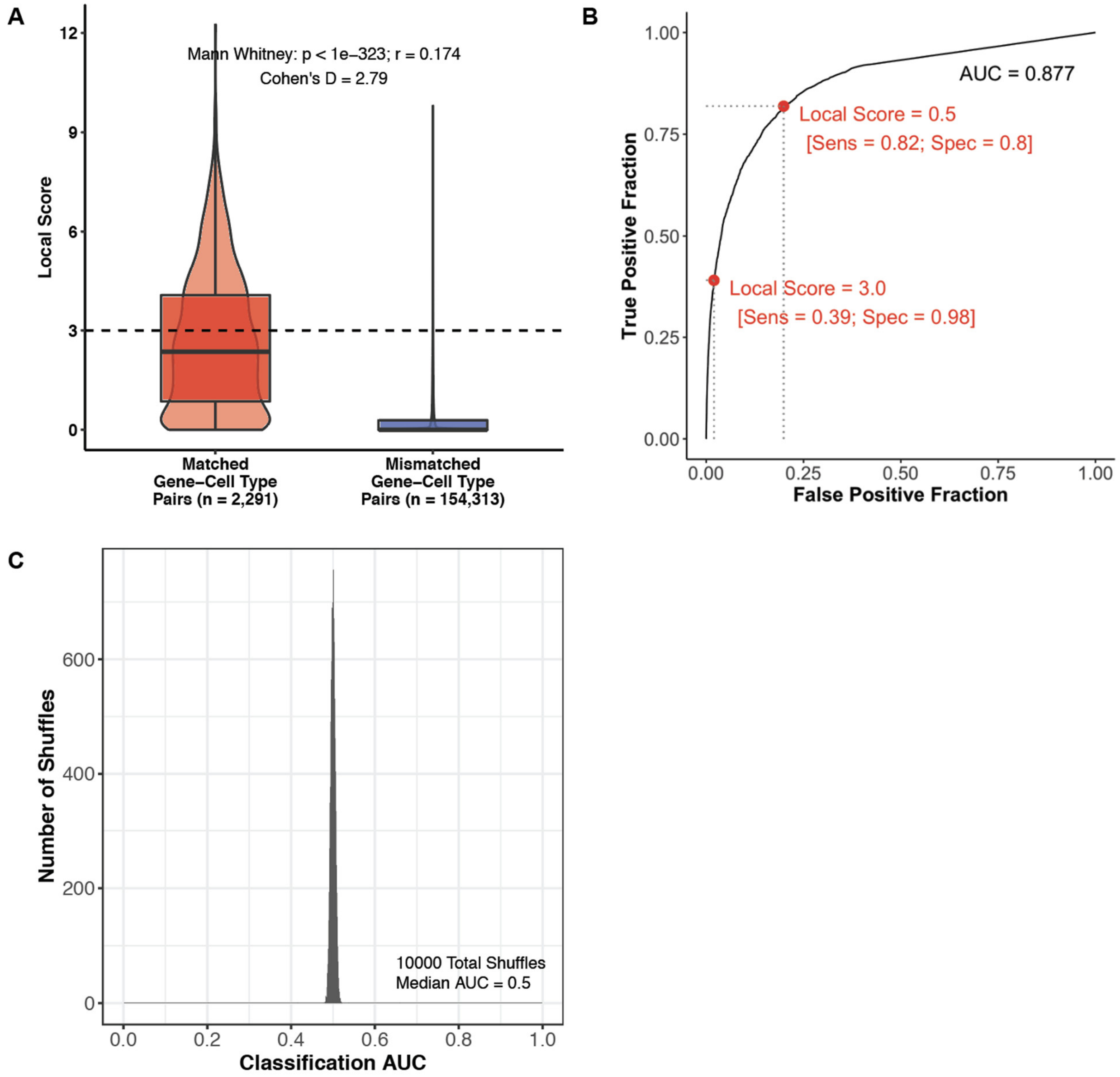

Figure S4. Literature based GCAs distinguish matched from mismatched gene-cell type pairs derived from the Panglao database of cell type markers. (A) Boxplot and violin plot showing the distribution of local scores (GCAs) between matched (n = 2,291) and mismatched (n = 154,313) gene-cell type pairs. The difference between these groups was assessed by calculating the Mann Whitney test p-value and effect size (r), along with the cohen's D effect size. (B) Receiver operating characteristic (ROC) analysis demonstrating the ability of literature based GCAs to classify matched versus mismatched gene-cell type pairs. The AUC was calculated as 0.877, and the sensitivity and specificity at specific local score thresholds are indicated in red. (C) Distribution of AUC values from 10,000 repeats of the analysis in (B), where the matched and mismatched labels were randomly shuffled prior to performing the ROC analysis. As expected, GCAs do not show any classification power when the labels are randomly assigned.

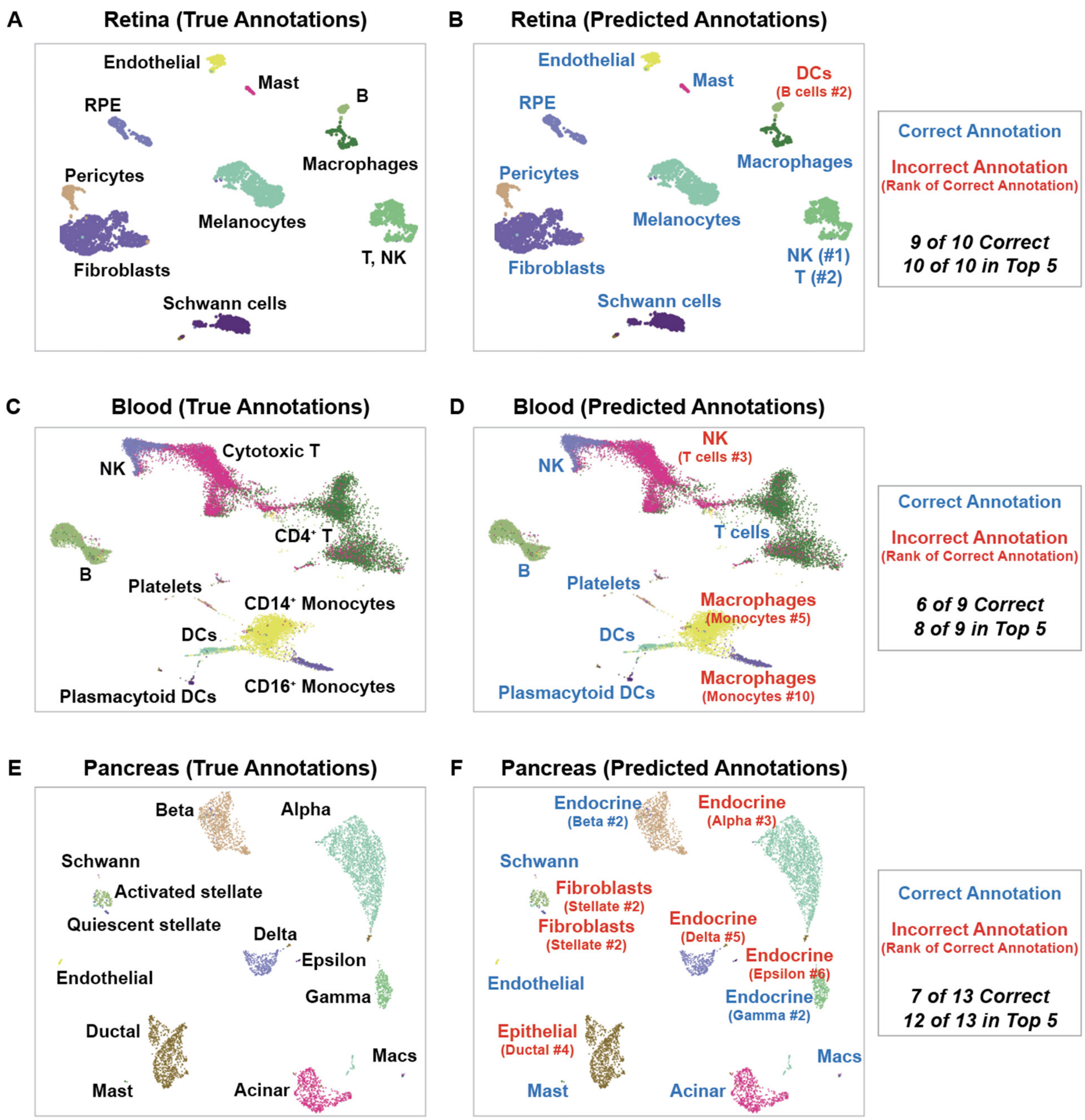

Figure S5. Comparison of true and predicted annotations for all cell types in three selected studies from retina, blood, and pancreas. True cluster annotations shown in the panels on the left (A, C, E) are derived from publicly deposited metadata and manual review. Predicted annotations on the right (B, D, F) are derived from scALE, with the optimized parameter settings as described in the text (pan-study reference, top 20 CDGs, scaled local score, weighting by log$_2$FC, and rank by L2 norm). Correct annotations (annotations in which the true priority node exactly matches the mapped priority node for the top prediction) are shown in green, and incorrect annotations are shown in red. For each incorrect annotation, the rank of the correct

prediction (out of 104 candidate cell type priority nodes) is shown in parentheses. The datasets selected for display here were all previously published in separate studies [1,2,4,5,9].

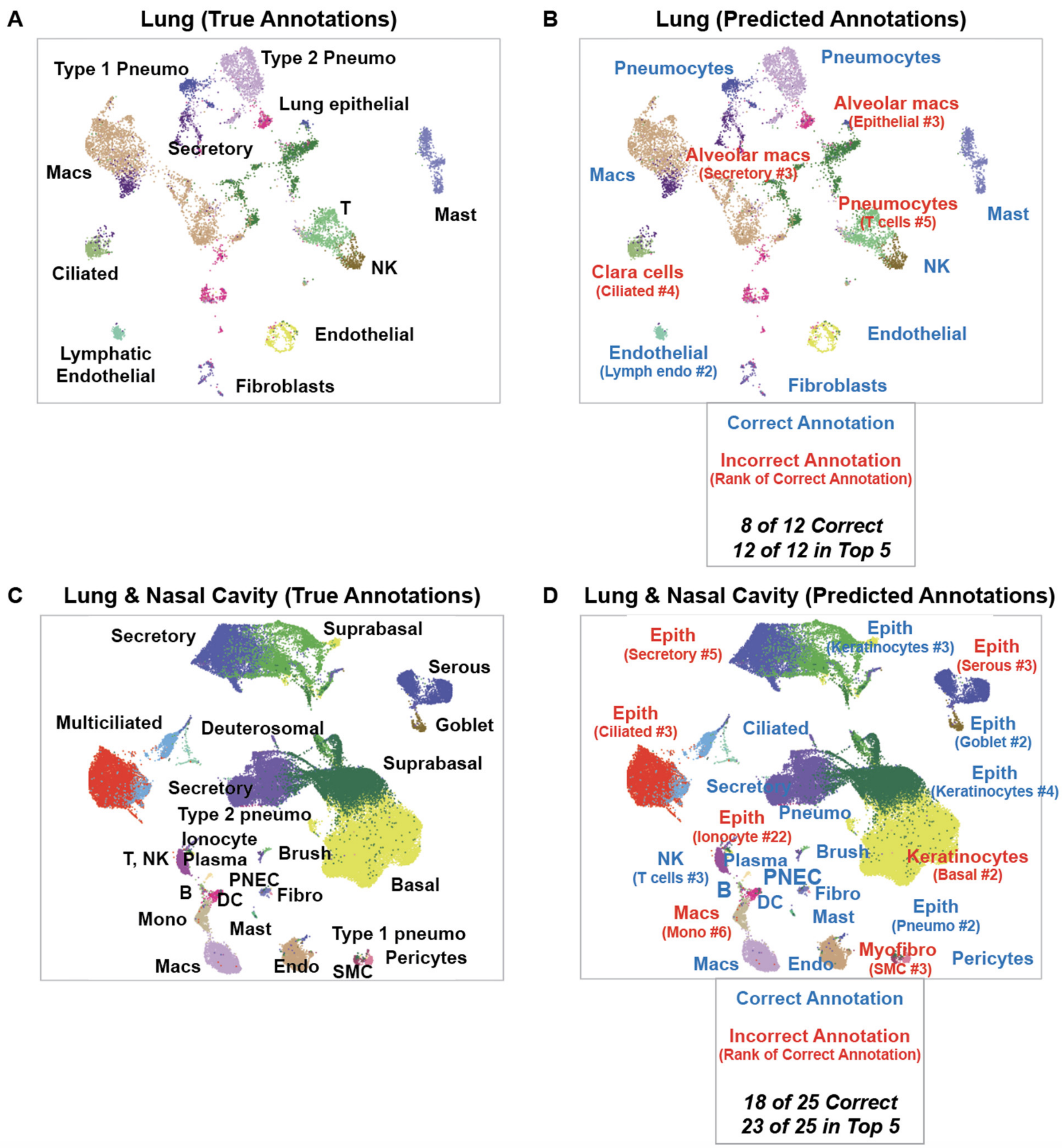

Figure S6. Comparison of true and predicted annotations for all cell types from two scRNA-seq studies of the respiratory tract (lung and nasal cavity). True cluster annotations shown in the panels on the left (A, C) are derived from publicly deposited metadata and manual review. Predicted annotations on the right (B, D) are derived from scALE, with the optimized parameter settings as described in the text (pan-study reference, top 20 CDGs, scaled local score, weighting by $\log_2$FC, and rank by L2 norm). Correct annotations (annotations in which the true priority node exactly matches the mapped priority node for the top prediction) are shown in green, and incorrect annotations are shown in red. For each incorrect annotation, the rank of the correct

prediction (out of 104 candidate cell type priority nodes) is shown in parentheses. The datasets selected for display here were previously published in separate studies [10,11].

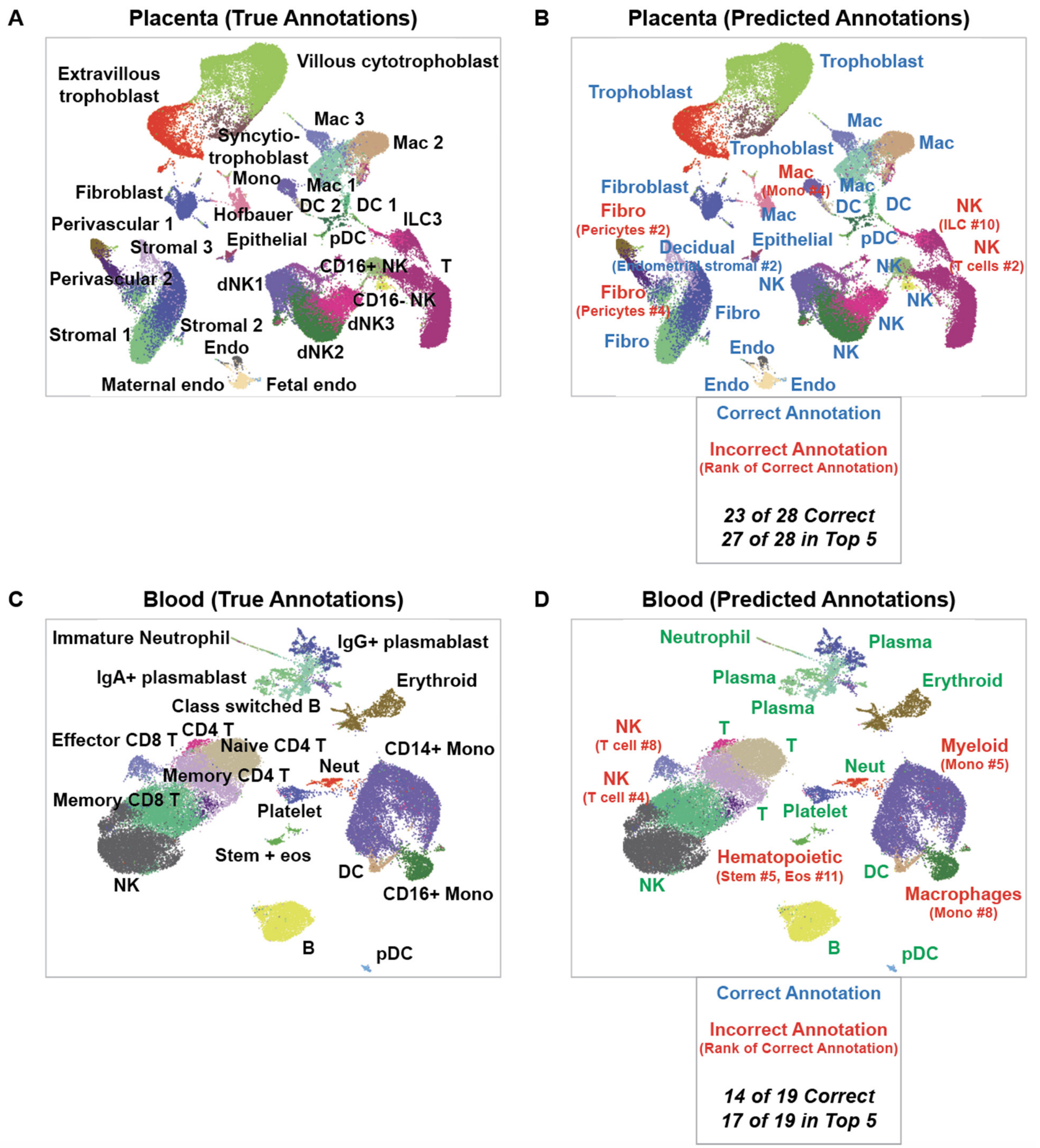

Figure S7. Comparison of true and predicted annotations for all cell types from scRNA-seq studies of the placenta and peripheral blood. True cluster annotations shown in the panels on the left (A, C) are derived from publicly deposited metadata and manual review. Predicted annotations on the right (B, D) are derived from scALE, with the optimized parameter settings as described in the text (pan-study reference, top 20 CDGs, scaled local score, weighting by log_2FC, and rank by L2 norm). Correct annotations (annotations in which the true priority node exactly matches the mapped priority node for the top prediction) are shown in green, and incorrect

annotations are shown in red. For each incorrect annotation, the rank of the correct prediction (out of 104 candidate cell type priority nodes) is shown in parentheses. The datasets selected for display here were previously published in separate studies [12,13].

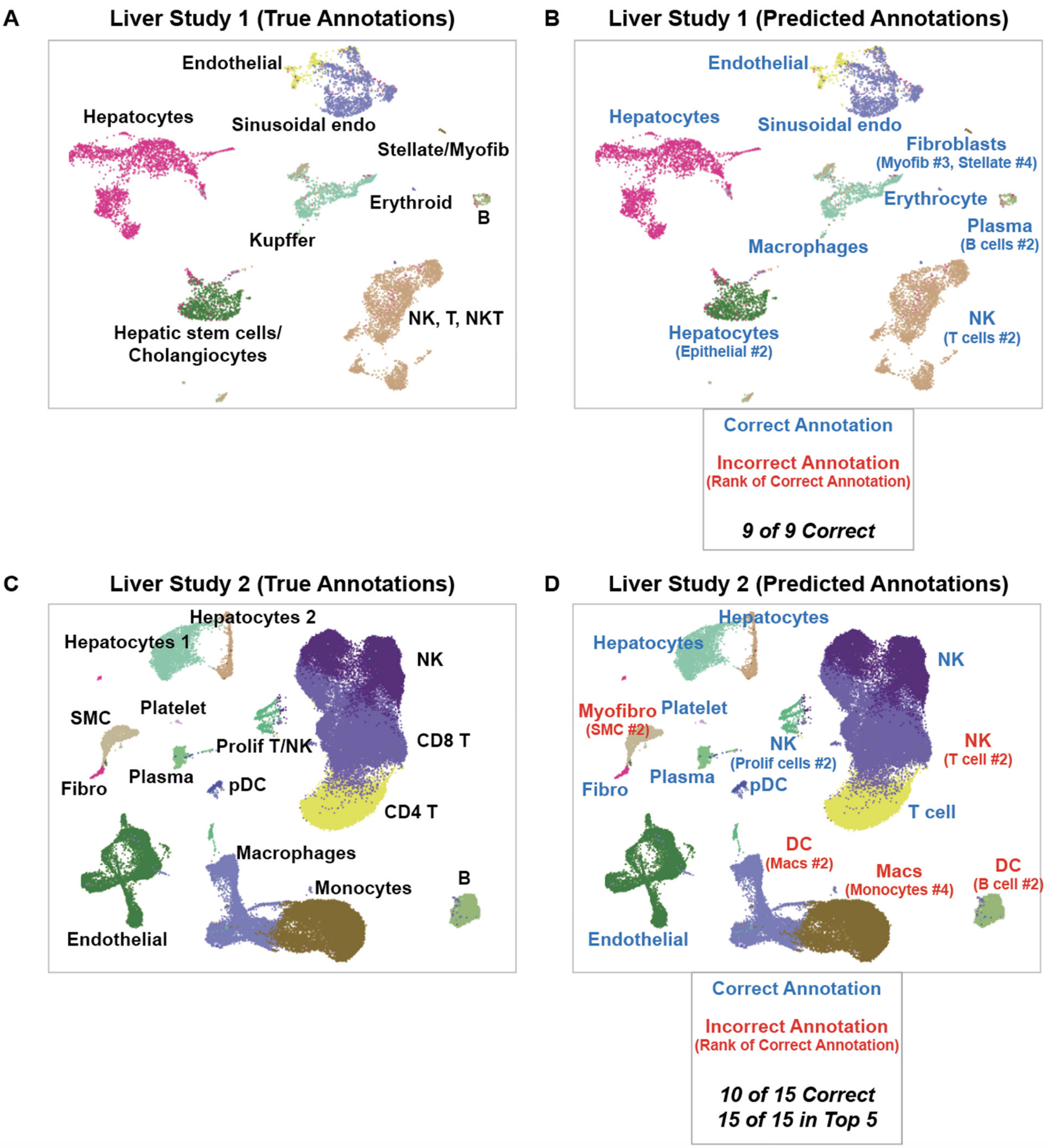

Figure S8. Comparison of true and predicted annotations for all cell types from two scRNA-seq studies of the human liver. True cluster annotations shown in the panels on the left (A, C) are derived from publicly deposited metadata and manual review. Predicted annotations on the right (B, D) are derived from scALE, with the optimized parameter settings as described in the text (pan-study reference, top 20 CDGs, scaled local score, weighting by $\log_2$FC, and rank by L2 norm). Correct annotations (annotations in which the true priority node exactly matches the mapped priority node for the top prediction) are shown in green, and incorrect annotations are shown in red. For each incorrect annotation, the rank of the correct prediction (out of 104

candidate cell type priority nodes) is shown in parentheses. The datasets selected for display here were previously published in separate studies [14,15].

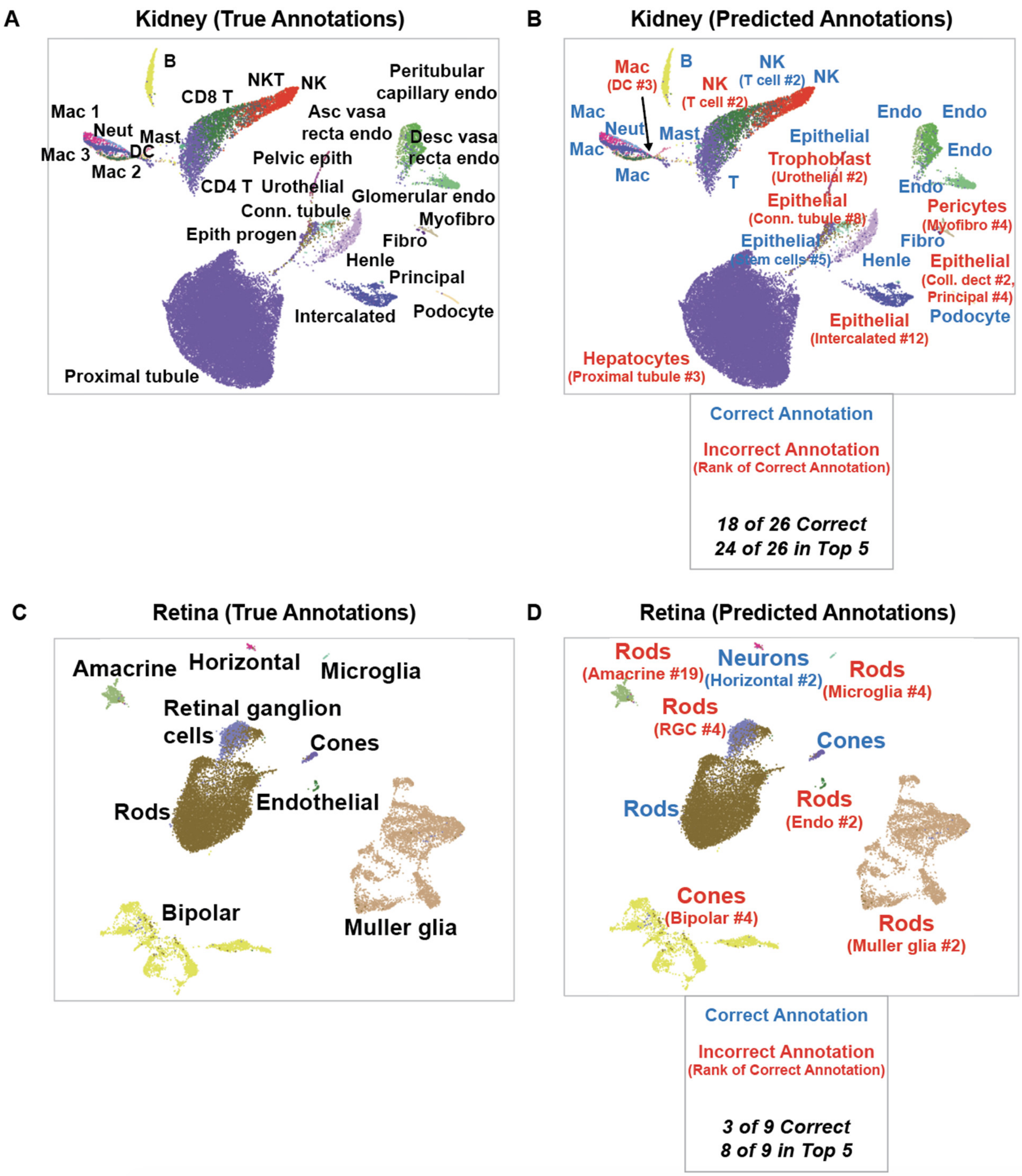

Figure S9. Comparison of true and predicted annotations for all cell types from scRNA-seq studies of the kidney and retina. True cluster annotations shown in the panels on the left (A, C) are derived from publicly deposited metadata and manual review. Predicted annotations on the right (B, D) are derived from scALE, with the optimized parameter settings as described in the text (pan-study reference, top 20 CDGs, scaled local score, weighting by log_2FC, and rank by L2 norm). Correct annotations (annotations in which the true priority node exactly matches the

mapped priority node for the top prediction) are shown in green, and incorrect annotations are shown in red. For each incorrect annotation, the rank of the correct prediction (out of 104 candidate cell type priority nodes) is shown in parentheses. The datasets selected for display here were previously published in separate studies [16,17].

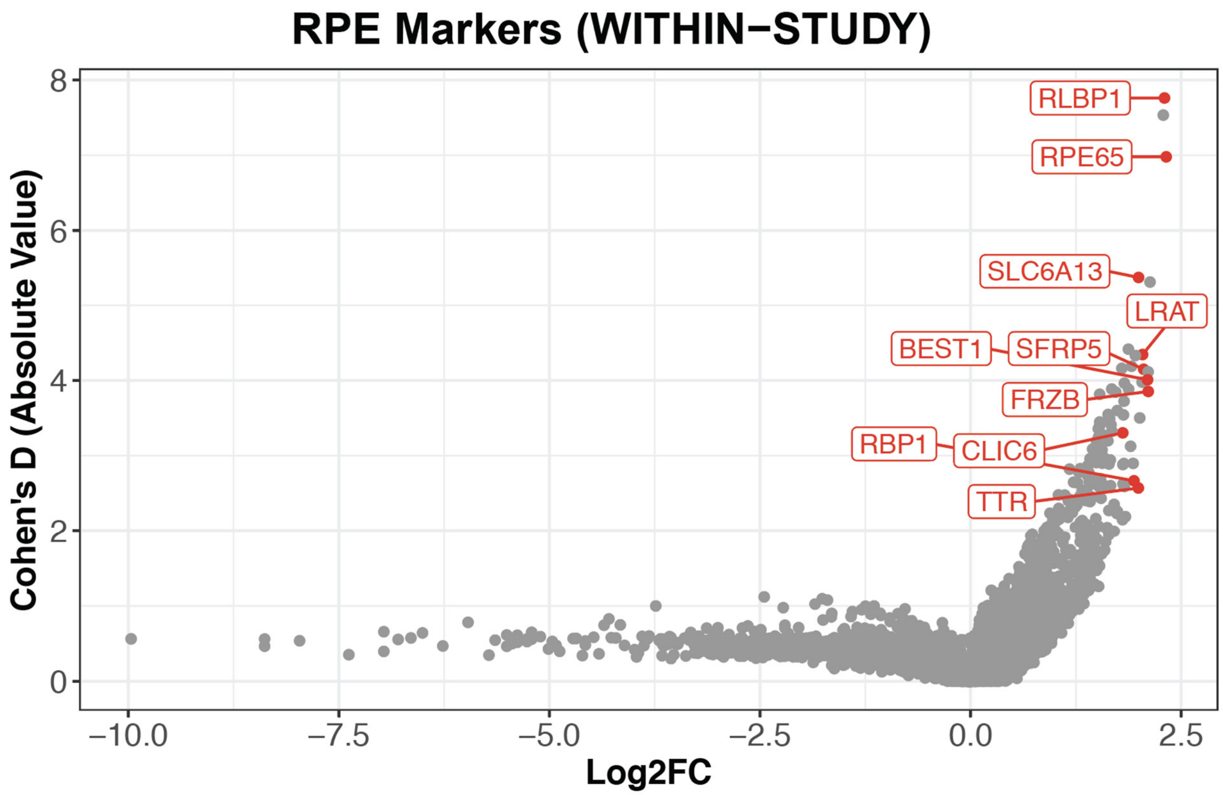

Figure S10. Confirmation of RPE marker genes using the "within-study" method of differential expression. To validate the candidate RPE markers which were identified using the "pan-study" approach (i.e., comparing the expression of all genes in RPE cells to the expression of all genes from all other studies in our entire reference dataset), we performed the more traditional "within-study" approach to calculate cluster defining genes. Specifically, we calculated the mean and standard deviation of expression (in units of CP10K) for all genes in each cluster identified in the previous study by Voigt, et al [4], and then calculated the fold change and cohen's D values by comparing the expression in the annotated RPE cells to the remaining cells in the dataset. The genes which were discussed in the text as RPE markers are highlighted in red.

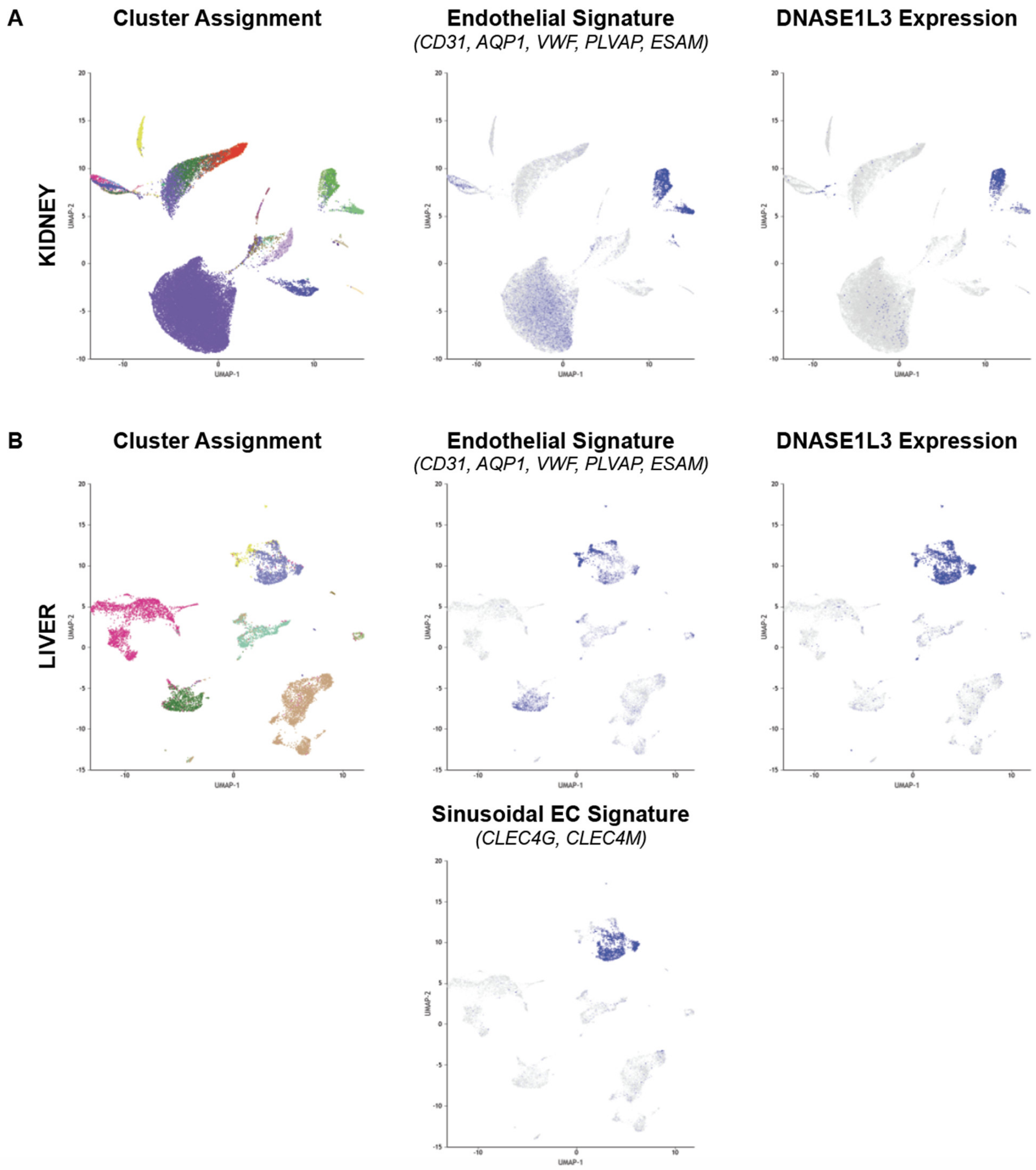

Figure S11. Expression of DNASE1L3 in endothelial cell populations from the kidney and liver. In each panel, the UMAP plot on the far left displays the clusters colored by their cell type annotations; the middle feature plot displays the expression level of a gene signature comprised of five canonical endothelial markers (CD31, AQP1, VWF, PLVAP, and ESAM) or two sinusoidal endothelial cell markers (CLEC4G and CLEC4M); and the far-right feature plot displays the expression level of DNASE1L3, which overlaps with the endothelial signatures in each case. The data is derived from (A) kidney [16] and (B) liver [14].

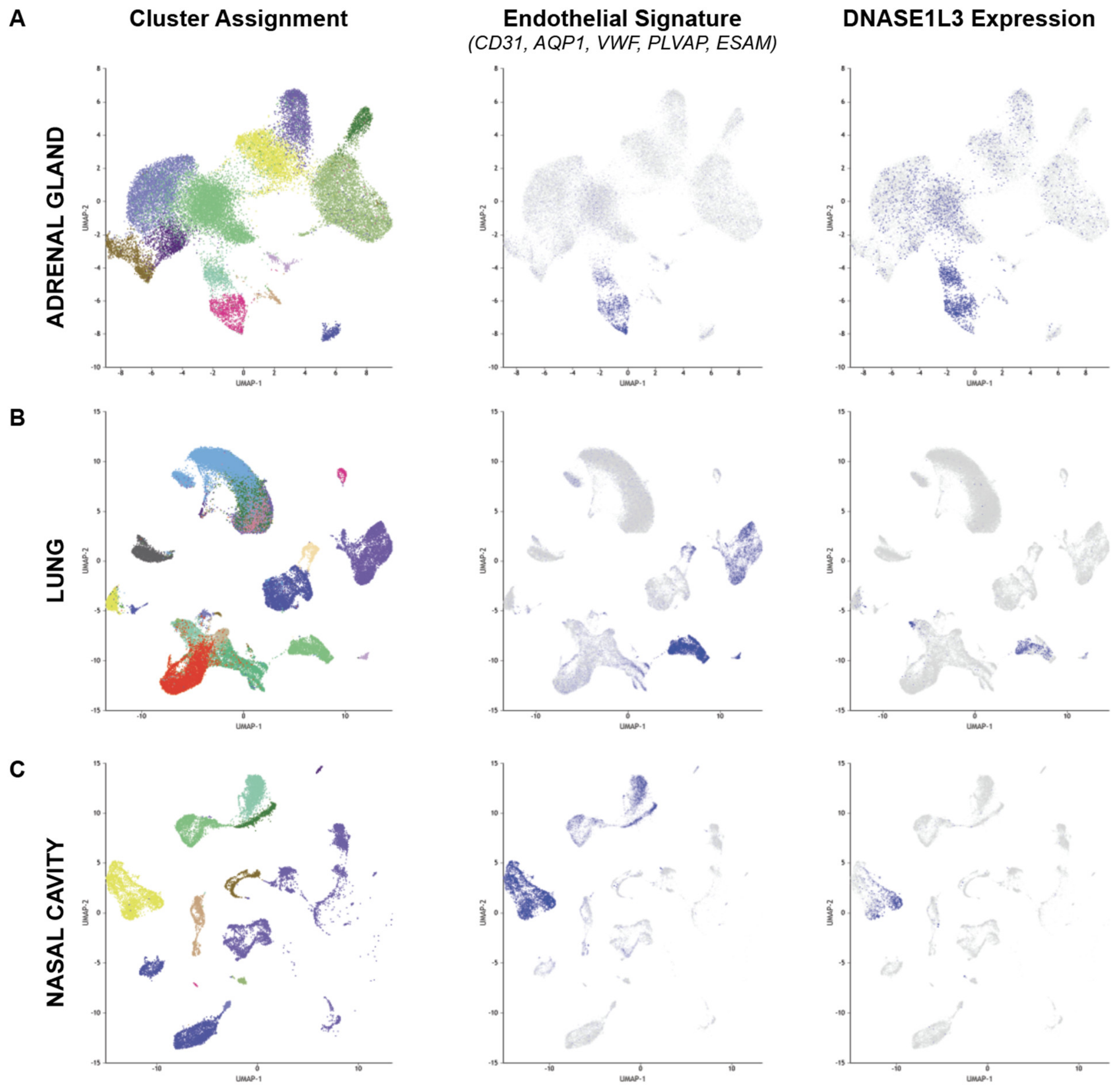

Figure S12. Expression of DNASE1L3 in endothelial cells from adrenal gland, lung, and nasal cavity. In each panel, the UMAP plot on the far left displays the clusters colored by their cell type annotations; the middle feature plot displays the expression level of a gene signature comprised of five canonical endothelial markers (CD31, AQP1, VWF, PLVAP, and ESAM); and the far-right feature plot displays the expression level of DNASE1L3, which overlaps with the endothelial signature in each case. The data is derived from (A) adrenal gland [18], (B) lung [19], and (c) nasal cavity [20].

Supplemental Tables

Table S1. Manually curated cell type defining genes. 174 gene-cell type pairs were extracted from published scRNA-seq datasets in which marker genes that were used for manual cluster annotation were reported [3,4,10,12–14,21,22]. This set of gene-cell type pairs were designated as "matched", and all other possible pairwise combinations of these genes and cell types were designated as "mismatched" pairs for subsequent analyses.

Cell Type	Marker Gene
ACINAR CELLS	PRSS1
ALPHA CELL	GCG
B CELL	CD37
B CELL	CD79A
B CELL	IGKC
B CELL	MS4A1
B CELL	MZB1
B CELL	PTPRC
BASAL CELL	KRT5
BASAL CELL	TP63
BRUSH CELLS	ASCL2
BRUSH CELLS	DCLK1
CD4 T CELLS	CCR7
CD4 T CELLS	IL7R
CD4 T CELLS	S100A4
CD8 T CELLS	CD8A
CHOLANGIOCYTES	CFTR
CHOLANGIOCYTES	EPCAM
CHOLANGIOCYTES	KRT17
CHOLANGIOCYTES	KRT18
CHOLANGIOCYTES	KRT19
CHOLANGIOCYTES	SOX9
CILIATED CELLS	FOXJ1
DENDRITIC CELLS	CCL17
DENDRITIC CELLS	CD1C
DENDRITIC CELLS	CD1E
DENDRITIC CELLS	CLEC10A

DENDRITIC CELLS	CST3
DENDRITIC CELLS	FCER1A
DUCTAL CELLS	KRT19
ENDOTHELIAL CELLS	ANGPT2
ENDOTHELIAL CELLS	CD34
ENDOTHELIAL CELLS	ICAM2
ENDOTHELIAL CELLS	PECAM1
ENDOTHELIAL CELLS	PLVAP
ENDOTHELIAL CELLS	VWF
EOSINOPHILS	CLC
EPSILON CELLS	GHRL
EXOCRINE CELLS	CCN3
EXOCRINE CELLS	RCAN1
EXOCRINE CELLS	SPP1
EXTRAVILLOUS TROPHOBLAST	HLA-E
EXTRAVILLOUS TROPHOBLAST	HLA-G
FIBROBLASTS	ACTA2
FIBROBLASTS	COL3A1
FIBROBLASTS	CYGB
FIBROBLASTS	FN1
FIBROBLASTS	IGF2
FIBROBLASTS	POSTN
FIBROBLASTS	TIMP1
GAMMA CELL	PPY
GAMMA DELTA T CELLS	TRDC
GAMMA DELTA T CELLS	TRGC1
GAMMA DELTA T CELLS	TRGC2
GOBLET CELLS	CEACAM5
GOBLET CELLS	MUC5AC
GOBLET CELLS	S100A4
HEPATIC STELLATE CELLS	CFTR
HEPATIC STELLATE CELLS	EPCAM
HEPATIC STELLATE CELLS	KRT17

HEPATIC STELLATE CELLS	KRT18
HEPATIC STELLATE CELLS	KRT19
HEPATIC STELLATE CELLS	SOX9
HEPATOCYTES	ALB
HEPATOCYTES	APOB
HEPATOCYTES	APOE
HEPATOCYTES	ASGR1
HEPATOCYTES	ASS1
HEPATOCYTES	CYP3A4
HEPATOCYTES	HP
HEPATOCYTES	PCK1
HEPATOCYTES	TF
IONOCYTES	CFTR
IONOCYTES	SCNN1B
KUPFFER CELLS	CD163
KUPFFER CELLS	MAFB
KUPFFER CELLS	VSIG4
LYMPHATIC ENDOTHELIAL CELLS	CCL21
MACROPHAGES	AIF1
MACROPHAGES	APOC1
MACROPHAGES	APOE
MACROPHAGES	CCL18
MACROPHAGES	CD14
MACROPHAGES	CD163
MACROPHAGES	CD68
MACROPHAGES	CD74
MACROPHAGES	CD86
MACROPHAGES	CSF1R
MACROPHAGES	HLA-DPA1
MACROPHAGES	HLA-DPB1
MACROPHAGES	HLA-DRA
MACROPHAGES	MAFB
MACROPHAGES	MARCO

MACROPHAGES	S100A9
MACROPHAGES	VSIG4
MAST CELLS	KIT
MAST CELLS	TPSAB1
MAST CELLS	TPSB2
MAST CELLS	TPSD1
MELANOCYTES	DCT
MELANOCYTES	MLANA
MELANOCYTES	PMEL
MELANOCYTES	TYRP1
MONOCYTES	CD14
MONOCYTES	FCGR3A
MONOCYTES	LYZ
MONOCYTES	MS4A7
NATURAL KILLER CELLS	CD2
NATURAL KILLER CELLS	CD9
NATURAL KILLER CELLS	FCGR3A
NATURAL KILLER CELLS	GNLY
NATURAL KILLER CELLS	ITGA1
NATURAL KILLER CELLS	KLRB1
NATURAL KILLER CELLS	NCAM1
NATURAL KILLER CELLS	NKG7
NATURAL KILLER CELLS	PRF1
NATURAL KILLER CELLS	PTPRC
NATURAL KILLER T CELLS	CD3E
NATURAL KILLER T CELLS	CD8A
NATURAL KILLER T CELLS	KLRB1
NATURAL KILLER T CELLS	PTPRC
NEUROENDOCRINE CELLS	ASCL1
NEUROENDOCRINE CELLS	CHGA
NEUROENDOCRINE CELLS	HOXB5
NEUROENDOCRINE CELLS	INSM1
NEUTROPHILS	CTSG

NEUTROPHILS	CXCR2
NEUTROPHILS	ELANE
NEUTROPHILS	FCGR3B
NEUTROPHILS	LCN2
NEUTROPHILS	LILRA5
NEUTROPHILS	LTF
NEUTROPHILS	MMP8
NEUTROPHILS	MPO
NEUTROPHILS	S100A12
NEUTROPHILS	S100A8
PANCREATIC BETA CELL	INS
PERIVASCULAR CELLS	MGP
PLATELET	PPBP
RETINAL PIGMENT EPITHELIAL CELLS	BEST1
RETINAL PIGMENT EPITHELIAL CELLS	RPE65
SCHWANN CELLS	PLP1
SINUSOIDAL ENDOTHELIAL CELLS	CLEC4G
SINUSOIDAL ENDOTHELIAL CELLS	CLEC4M
SINUSOIDAL ENDOTHELIAL CELLS	FLT1
SMOOTH MUSCLE CELLS	ACTA2
SMOOTH MUSCLE CELLS	RGS5
STELLATE CELLS	ACTA2
STELLATE CELLS	COL3A1
STELLATE CELLS	CYGB
STELLATE CELLS	FN1
STELLATE CELLS	POSTN
STELLATE CELLS	TIMP1
STEM CELLS	CD34
STROMAL CELLS	ACTA2
STROMAL CELLS	COL3A1
STROMAL CELLS	CYGB
STROMAL CELLS	DKK1
STROMAL CELLS	FN1

STROMAL CELLS	IGFBP1
STROMAL CELLS	IGFBP2
STROMAL CELLS	IGFBP6
STROMAL CELLS	POSTN
STROMAL CELLS	TAGLN
STROMAL CELLS	TIMP1
T CELL	CCR7
T CELL	CD2
T CELL	CD3D
T CELL	CD3E
T CELL	CD8A
T CELL	IL7R
T CELL	KLRB1
T CELL	PTPRC
T CELL	S100A4

Table S2. Summary of annotation algorithm performance among tuning studies for all tested parameter combinations. The "Parameter Settings" column indicates the combination of parameters which was used to annotate clusters, including the following listed in this order: (1) local score version (raw or scaled), (2) reference cells used to calculate CDGs (pan-study, within tissue, or within study), (3) number of CDGs used (1, 3, 5, 10, or 20), (4) the weighting method used calculating L2 norms (unweighted, fold change, or log_2FC), and (5) the ranking metric used (modified L0 rank, L2 rank, or composite rank). The next five columns indicate the percentage of clusters (out of 185) for which the correct annotation was among the top-ranked 1, 2, 3, 4, or 5 predictions. The last column provides the $AUC_{Ranks\ 1\text{-}5}$ metric for each parameter combination, which was calculated as the average of the previous five columns. The table is sorted in descending order by $AUC_{Ranks\ 1\text{-}5}$, which was used to assess overall algorithm performance.

Parameter Settings	% Clusters Annotated Correctly (Rank 1)	% Clusters Annotated Correctly (Ranks 1-2)	% Clusters Annotated Correctly (Ranks 1-3)	% Clusters Annotated Correctly (Ranks 1-4)	% Clusters Annotated Correctly (Ranks 1-5)	$AUC_{Ranks\ 1\text{-}5}$
Scaled_PanStudy_Top20CDGs_WeightLog2FC_L2Rank	66.49	78.92	84.32	90.27	93.51	82.7
Scaled_WithinStudy_Top20CDGs_WeightLog2FC_L2Rank	65.95	80.54	84.32	89.19	91.89	82.38
Scaled_PanStudy_Top20CDGs_Unweighted_L2Rank	63.78	76.22	81.08	90.27	90.81	80.43
Scaled_PanStudy_Top10CDGs_WeightLog2FC_L2Rank	64.86	75.68	83.78	87.57	89.73	80.32
Scaled_WithinStudy_Top10CDGs_WeightLog2FC_L2Rank	61.08	72.43	83.78	89.19	92.43	79.78
Scaled_WithinStudy_Top20CDGs_Unweighted_L2Rank	62.16	77.3	81.62	87.03	89.73	79.57
Scaled_WithinTissue_Top20CDGs_WeightLog2FC_L2Rank	61.62	76.22	82.7	87.03	89.19	79.35
Scaled_PanStudy_Top20CDGs_WeightFC_CompositeRank	62.7	75.68	82.16	84.86	88.65	78.81
Raw_PanStudy_Top10CDGs_WeightFC_CompositeRank	62.16	76.22	81.62	87.03	87.03	78.81
Scaled_PanStudy_Top10CDGs_Unweighted_L2Rank	63.24	73.51	83.78	85.95	87.03	78.7
Scaled_WithinStudy_Top10CDGs_Unweighted_L2Rank	58.92	71.35	82.7	88.65	90.81	78.49
Raw_PanStudy_Top10CDGs_WeightLog2FC_L2Rank	63.24	73.51	81.08	85.95	88.11	78.38
Raw_PanStudy_Top20CDGs_WeightFC_CompositeRank	62.7	74.59	81.08	85.95	87.03	78.27
Scaled_PanStudy_Top20CDGs_WeightFC_L2Rank	65.41	74.05	82.16	83.24	85.95	78.16
Scaled_PanStudy_Top20CDGs_WeightLog2FC_CompositeRank	58.38	75.14	81.62	87.03	88.11	78.06

**Raw_PanStudy_Top10CDGs_WeightLog2FC_CompositeRank**	61.62	75.68	81.08	84.32	87.57	78.05
**Scaled_PanStudy_Top10CDGs_WeightLog2FC_CompositeRank**	62.7	73.51	81.62	83.78	88.11	77.94
**Raw_PanStudy_Top10CDGs_Unweighted_CompositeRank**	61.62	76.22	80	83.24	87.57	77.73
**Scaled_WithinStudy_Top20CDGs_WeightFC_L2Rank**	60	72.97	82.16	85.41	88.11	77.73
**Raw_WithinTissue_Top10CDGs_WeightFC_L2Rank**	60.54	74.59	80.54	84.86	87.57	77.62
**Raw_WithinTissue_Top20CDGs_WeightFC_L2Rank**	60.54	74.59	80	83.78	89.19	77.62
**Raw_PanStudy_Top20CDGs_WeightFC_L2Rank**	62.16	72.97	80	84.86	87.57	77.51
**Scaled_PanStudy_Top10CDGs_WeightFC_CompositeRank**	62.16	74.59	79.46	83.78	87.57	77.51
**Scaled_WithinTissue_Top20CDGs_WeightFC_L2Rank**	61.08	71.89	80.54	85.41	88.65	77.51
**Scaled_WithinStudy_Top5CDGs_Unweighted_L2Rank**	60	72.43	80	86.49	88.65	77.51
**Scaled_WithinTissue_Top10CDGs_WeightLog2FC_L2Rank**	60	70.81	81.08	85.95	89.19	77.41
**Scaled_WithinTissue_Top20CDGs_Unweighted_L2Rank**	60.54	74.59	80.54	84.86	86.49	77.4
**Scaled_WithinStudy_Top10CDGs_WeightFC_CompositeRank**	61.08	72.43	78.92	85.95	88.11	77.3
**Scaled_WithinStudy_Top10CDGs_WeightLog2FC_CompositeRank**	61.08	71.89	80	86.49	87.03	77.3
**Scaled_WithinStudy_Top10CDGs_Unweighted_CompositeRank**	62.16	71.89	78.92	85.95	87.03	77.19
**Raw_WithinStudy_Top5CDGs_Unweighted_L2Rank**	57.84	71.89	82.16	85.41	88.65	77.19
**Scaled_WithinStudy_Top5CDGs_WeightLog2FC_L2Rank**	61.08	70.81	81.08	85.41	87.03	77.08
**Raw_PanStudy_Top10CDGs_WeightFC_L2Rank**	59.46	71.89	81.08	85.41	87.57	77.08
**Scaled_WithinStudy_Top10CDGs_WeightFC_L2Rank**	56.76	71.89	81.62	85.41	89.73	77.08
**Scaled_PanStudy_Top20CDGs_Unweighted_CompositeRank**	59.46	72.43	81.08	85.41	85.95	76.87
**Raw_WithinTissue_Top10CDGs_WeightLog2FC_L2Rank**	58.92	74.59	80	83.24	87.57	76.86
**Raw_PanStudy_Top10CDGs_Unweighted_L2Rank**	62.16	73.51	78.92	82.7	86.49	76.76
**Scaled_WithinStudy_Top20CDGs_WeightFC_CompositeRank**	61.08	71.89	78.92	84.86	87.03	76.76
**Raw_WithinStudy_Top10CDGs_WeightLog2FC_L2Rank**	57.84	74.05	80	83.78	88.11	76.76
**Scaled_WithinStudy_Top5CDGs_W**	60	71.89	78.92	85.41	87.03	76.65

eightFC_CompositeRank						
Raw_PanStudy_Top5CDGs_WeightLog2FC_L2Rank	58.92	71.35	80.54	85.95	86.49	76.65
Scaled_WithinTissue_Top20CDGs_WeightFC_CompositeRank	60.54	71.89	80	83.78	86.49	76.54
Scaled_WithinStudy_Top20CDGs_WeightLog2FC_CompositeRank	59.46	68.65	80.54	85.95	87.57	76.43
Scaled_WithinStudy_Top20CDGs_Unweighted_CompositeRank	60	68.65	79.46	85.95	87.57	76.33
Scaled_PanStudy_Top10CDGs_Unweighted_CompositeRank	62.16	71.89	78.92	83.24	85.41	76.32
Raw_WithinStudy_Top5CDGs_WeightLog2FC_L2Rank	56.76	70.81	81.08	84.86	88.11	76.32
Raw_WithinStudy_Top10CDGs_WeightFC_CompositeRank	61.08	70.81	78.92	82.16	88.11	76.22
Raw_WithinStudy_Top10CDGs_WeightLog2FC_CompositeRank	62.16	70.81	78.38	82.7	86.49	76.11
Scaled_PanStudy_Top10CDGs_WeightFC_L2Rank	61.62	71.89	78.38	82.7	85.95	76.11
Raw_WithinStudy_Top20CDGs_WeightFC_L2Rank	60	73.51	78.38	82.16	86.49	76.11
Scaled_WithinTissue_Top10CDGs_WeightLog2FC_CompositeRank	62.16	69.19	78.38	83.24	87.03	76
Raw_WithinStudy_Top10CDGs_Unweighted_CompositeRank	62.16	71.89	78.38	82.16	85.41	76
Raw_WithinStudy_Top10CDGs_WeightFC_L2Rank	58.92	73.51	79.46	82.16	85.95	76
Raw_PanStudy_Top5CDGs_Unweighted_L2Rank	58.38	71.35	79.46	84.86	85.95	76
Raw_WithinStudy_Top5CDGs_Unweighted_CompositeRank	57.84	71.35	79.46	83.78	87.57	76
Scaled_WithinTissue_Top20CDGs_Unweighted_CompositeRank	60	71.89	78.38	83.24	85.95	75.89
Scaled_WithinTissue_Top10CDGs_Unweighted_L2Rank	56.22	70.81	80	85.41	87.03	75.89
Scaled_PanStudy_Top5CDGs_WeightLog2FC_L2Rank	61.62	70.27	80	81.08	85.41	75.68
Scaled_WithinTissue_Top20CDGs_WeightLog2FC_CompositeRank	60	70.81	78.38	83.78	85.41	75.68
Raw_WithinStudy_Top5CDGs_WeightLog2FC_CompositeRank	57.3	70.81	79.46	82.7	88.11	75.68
Scaled_WithinTissue_Top10CDGs_Unweighted_CompositeRank	61.62	68.65	77.84	83.78	85.95	75.57
Raw_WithinTissue_Top10CDGs_WeightFC_CompositeRank	61.08	70.81	76.76	83.24	85.41	75.46
Raw_PanStudy_Top20CDGs_WeightLog2FC_CompositeRank	60.54	72.97	78.38	81.08	84.32	75.46
Raw_WithinStudy_Top5CDGs_WeightFC_CompositeRank	57.3	71.35	80	82.7	85.95	75.46

**Raw_WithinTissue_Top20CDGs_WeightFC_CompositeRank**	61.62	73.51	77.84	79.46	84.32	75.35
**Raw_WithinTissue_Top10CDGs_WeightLog2FC_CompositeRank**	61.08	70.81	77.3	81.62	85.95	75.35
**Scaled_WithinTissue_Top10CDGs_WeightFC_CompositeRank**	61.08	68.65	76.22	84.32	86.49	75.35
**Raw_WithinTissue_Top20CDGs_WeightLog2FC_L2Rank**	60	75.14	78.92	80	82.7	75.35
**Scaled_WithinStudy_Top5CDGs_WeightLog2FC_CompositeRank**	58.92	70.81	75.14	84.86	87.03	75.35
**Scaled_WithinStudy_Top5CDGs_WeightFC_L2Rank**	56.76	70.27	78.92	83.24	87.57	75.35
**Raw_PanStudy_Top20CDGs_WeightLog2FC_L2Rank**	61.08	71.35	77.3	81.62	84.86	75.24
**Scaled_WithinStudy_Top5CDGs_Unweighted_CompositeRank**	59.46	71.35	75.68	82.7	87.03	75.24
**Scaled_WithinTissue_Top10CDGs_WeightFC_L2Rank**	56.22	70.27	77.84	84.32	87.57	75.24
**Raw_WithinStudy_Top20CDGs_WeightLog2FC_L2Rank**	57.84	71.35	78.38	83.24	84.86	75.13
**Raw_WithinTissue_Top10CDGs_Unweighted_L2Rank**	57.84	71.89	78.38	81.08	85.95	75.03
**Raw_PanStudy_Top5CDGs_WeightFC_CompositeRank**	57.3	69.73	78.92	83.24	84.86	74.81
**Raw_WithinTissue_Top10CDGs_Unweighted_CompositeRank**	60.54	70.27	76.22	81.62	84.86	74.7
**Raw_WithinStudy_Top10CDGs_Unweighted_L2Rank**	57.3	71.89	78.38	81.62	84.32	74.7
**Raw_PanStudy_Top20CDGs_Unweighted_CompositeRank**	59.46	71.89	78.38	80	82.7	74.49
**Scaled_PanStudy_Top5CDGs_WeightFC_CompositeRank**	58.92	69.73	77.84	81.62	84.32	74.49
**Raw_PanStudy_Top5CDGs_WeightFC_L2Rank**	55.14	69.73	78.38	83.78	85.41	74.49
**Raw_PanStudy_Top5CDGs_WeightLog2FC_CompositeRank**	57.84	68.65	78.38	82.7	84.32	74.38
**Scaled_PanStudy_Top5CDGs_Unweighted_L2Rank**	59.46	69.19	77.84	80.54	84.32	74.27
**Raw_PanStudy_Top5CDGs_Unweighted_CompositeRank**	57.84	69.73	78.38	81.62	83.78	74.27
**Raw_WithinTissue_Top5CDGs_WeightLog2FC_L2Rank**	55.14	70.27	77.3	81.62	85.95	74.06
**Raw_WithinStudy_Top5CDGs_WeightFC_L2Rank**	53.51	71.35	78.38	82.7	84.32	74.05
**Raw_WithinTissue_Top20CDGs_WeightLog2FC_CompositeRank**	60	73.51	76.22	78.92	81.08	73.95
**Scaled_PanStudy_Top5CDGs_WeightLog2FC_CompositeRank**	57.84	68.11	77.3	81.62	84.86	73.95
**Raw_WithinStudy_Top20CDGs_We**	57.84	68.11	77.84	81.08	83.78	73.73

ightFC_CompositeRank						
Raw_WithinTissue_Top20CDGs_Unweighted_CompositeRank	60.54	72.97	75.68	77.84	81.08	73.62
Raw_WithinTissue_Top5CDGs_WeightFC_L2Rank	55.14	70.81	76.76	80	85.41	73.62
Raw_WithinTissue_Top20CDGs_Unweighted_L2Rank	58.92	72.43	77.3	78.38	80.54	73.51
Scaled_PanStudy_Top5CDGs_Unweighted_CompositeRank	57.84	67.57	76.76	81.08	83.78	73.41
Raw_WithinTissue_Top5CDGs_Unweighted_L2Rank	55.14	69.19	76.76	80	85.95	73.41
Raw_WithinStudy_Top20CDGs_WeightLog2FC_CompositeRank	58.38	68.65	76.22	79.46	83.24	73.19
Raw_WithinStudy_Top3CDGs_WeightLog2FC_L2Rank	52.97	71.35	77.84	81.08	82.7	73.19
Raw_PanStudy_Top20CDGs_Unweighted_L2Rank	57.84	70.27	76.22	78.92	82.16	73.08
Raw_PanStudy_Top3CDGs_Unweighted_L2Rank	52.43	67.57	77.84	83.24	84.32	73.08
Raw_WithinStudy_Top20CDGs_Unweighted_L2Rank	58.38	69.73	76.22	79.46	81.08	72.97
Raw_PanStudy_Top3CDGs_WeightLog2FC_L2Rank	51.89	68.11	77.3	82.7	84.86	72.97
Scaled_PanStudy_Top5CDGs_WeightFC_L2Rank	57.3	65.95	75.68	81.08	83.78	72.76
Raw_WithinStudy_Top20CDGs_Unweighted_CompositeRank	58.38	68.65	75.14	78.38	82.7	72.65
Scaled_WithinTissue_Top5CDGs_WeightLog2FC_L2Rank	54.59	64.86	77.3	81.08	85.41	72.65
Raw_PanStudy_Top3CDGs_Unweighted_CompositeRank	54.05	65.95	77.84	82.16	83.24	72.65
Raw_WithinTissue_Top3CDGs_WeightLog2FC_CompositeRank	52.97	66.49	76.76	83.24	83.78	72.65
Raw_WithinTissue_Top5CDGs_WeightFC_CompositeRank	54.59	70.27	74.59	78.92	84.32	72.54
Raw_PanStudy_Top3CDGs_WeightLog2FC_CompositeRank	54.05	66.49	77.3	81.62	83.24	72.54
Raw_WithinTissue_Top3CDGs_Unweighted_CompositeRank	52.97	67.03	76.76	82.16	83.78	72.54
Raw_WithinTissue_Top3CDGs_WeightLog2FC_L2Rank	52.43	68.65	76.76	81.08	83.24	72.43
Raw_WithinTissue_Top5CDGs_WeightLog2FC_CompositeRank	54.05	68.11	74.05	80.54	84.86	72.32
Scaled_WithinTissue_Top5CDGs_Unweighted_L2Rank	54.05	64.86	75.68	81.08	85.41	72.22
Raw_WithinStudy_Top3CDGs_Unweighted_L2Rank	52.43	69.73	76.76	80	82.16	72.22
Raw_WithinTissue_Top3CDGs_Unweighted_L2Rank	51.89	69.19	75.68	81.08	83.24	72.22

**Raw_WithinTissue_Top5CDGs_Unweighted_CompositeRank**	54.59	67.57	73.51	79.46	85.41	72.11
**Raw_WithinStudy_Top3CDGs_WeightLog2FC_CompositeRank**	53.51	68.65	74.59	80	82.7	71.89
**Raw_WithinStudy_Top3CDGs_WeightFC_L2Rank**	51.89	67.03	77.84	81.08	81.62	71.89
**Raw_PanStudy_Top3CDGs_WeightFC_CompositeRank**	52.97	65.95	76.22	81.08	82.16	71.68
**Scaled_WithinTissue_Top5CDGs_WeightLog2FC_CompositeRank**	54.05	63.78	73.51	81.62	84.32	71.46
**Raw_WithinStudy_Top3CDGs_WeightFC_CompositeRank**	52.43	67.03	75.68	80.54	81.62	71.46
**Raw_PanStudy_Top20CDGs_Unweighted_L0Rank**	55.14	67.57	75.68	77.84	80.54	71.35
**Raw_WithinTissue_Top3CDGs_WeightFC_CompositeRank**	51.89	65.41	73.51	81.62	84.32	71.35
**Scaled_WithinTissue_Top5CDGs_Unweighted_CompositeRank**	54.05	63.78	73.51	80	84.86	71.24
**Raw_WithinStudy_Top3CDGs_Unweighted_CompositeRank**	52.97	67.57	74.05	78.92	82.16	71.13
**Raw_PanStudy_Top3CDGs_WeightFC_L2Rank**	50.27	66.49	76.22	80.54	81.62	71.03
**Scaled_WithinTissue_Top5CDGs_WeightFC_CompositeRank**	53.51	62.16	72.97	80.54	84.86	70.81
**Scaled_WithinTissue_Top5CDGs_WeightFC_L2Rank**	50.81	62.7	75.68	80	83.24	70.49
**Raw_WithinTissue_Top3CDGs_WeightFC_L2Rank**	49.19	67.03	74.59	77.84	83.24	70.38
**Scaled_PanStudy_Top3CDGs_WeightFC_CompositeRank**	55.14	62.16	72.43	77.84	82.16	69.95
**Scaled_PanStudy_Top3CDGs_WeightLog2FC_CompositeRank**	55.68	62.16	71.35	76.76	82.16	69.62
**Scaled_WithinStudy_Top3CDGs_WeightFC_CompositeRank**	52.43	64.86	72.43	76.76	81.62	69.62
**Scaled_PanStudy_Top3CDGs_Unweighted_CompositeRank**	55.68	63.24	70.27	76.22	82.16	69.51
**Scaled_WithinTissue_Top3CDGs_WeightLog2FC_CompositeRank**	49.73	61.62	75.14	78.92	81.08	69.3
**Scaled_WithinStudy_Top3CDGs_WeightLog2FC_CompositeRank**	50.81	65.41	72.97	76.76	80	69.19
**Scaled_PanStudy_Top3CDGs_Unweighted_L2Rank**	52.43	64.32	72.43	76.22	80	69.08
**Scaled_WithinTissue_Top3CDGs_Unweighted_CompositeRank**	49.19	62.16	74.05	78.92	80.54	68.97
**Scaled_PanStudy_Top3CDGs_WeightLog2FC_L2Rank**	50.81	64.86	72.43	76.76	79.46	68.86
**Scaled_WithinStudy_Top3CDGs_Unweighted_CompositeRank**	50.81	64.86	71.89	76.76	80	68.86
**Scaled_WithinTissue_Top3CDGs_**	50.27	61.62	73.51	77.84	81.08	68.86

**WeightFC_CompositeRank**						
**Raw_WithinTissue_Top20CDGs_Unweighted_L0Rank**	56.76	65.41	70.27	74.05	77.3	68.76
**Scaled_WithinStudy_Top3CDGs_WeightLog2FC_L2Rank**	48.11	63.24	72.97	77.84	80	68.43
**Scaled_WithinStudy_Top3CDGs_Unweighted_L2Rank**	48.11	62.16	72.97	78.38	80	68.32
**Raw_WithinStudy_Top20CDGs_Unweighted_L0Rank**	55.68	63.78	68.65	74.05	75.68	67.57
**Scaled_WithinTissue_Top3CDGs_WeightLog2FC_L2Rank**	49.73	61.08	71.35	76.76	78.92	67.57
**Raw_PanStudy_Top10CDGs_Unweighted_L0Rank**	50.27	63.78	70.81	75.14	77.3	67.46
**Scaled_WithinStudy_Top3CDGs_WeightFC_L2Rank**	48.65	62.16	70.81	75.68	79.46	67.35
**Scaled_PanStudy_Top3CDGs_WeightFC_L2Rank**	50.81	61.62	69.73	75.68	78.38	67.24
**Raw_WithinStudy_Top10CDGs_Unweighted_L0Rank**	50.81	62.16	69.73	75.68	77.84	67.24
**Scaled_WithinTissue_Top3CDGs_Unweighted_L2Rank**	49.19	60.54	70.81	77.3	78.38	67.24
**Raw_WithinTissue_Top10CDGs_Unweighted_L0Rank**	53.51	63.24	68.11	73.51	76.76	67.03
**Scaled_WithinTissue_Top3CDGs_WeightFC_L2Rank**	47.57	60	69.73	75.68	77.84	66.16
**Raw_WithinStudy_Top1CDG_WeightFC_CompositeRank**	40	58.38	65.41	69.73	72.97	61.3
**Raw_WithinStudy_Top1CDG_WeightFC_L2Rank**	40	58.38	65.41	69.73	72.97	61.3
**Raw_WithinStudy_Top1CDG_WeightLog2FC_CompositeRank**	40	58.38	65.41	69.73	72.97	61.3
**Raw_WithinStudy_Top1CDG_WeightLog2FC_L2Rank**	40	58.38	65.41	69.73	72.97	61.3
**Raw_WithinStudy_Top1CDG_Unweighted_CompositeRank**	40	58.38	65.41	69.73	72.97	61.3
**Raw_WithinStudy_Top1CDG_Unweighted_L2Rank**	40	58.38	65.41	69.73	72.97	61.3
**Raw_WithinStudy_Top5CDGs_Unweighted_L0Rank**	36.76	54.59	65.41	69.19	71.35	59.46
**Scaled_WithinStudy_Top1CDG_WeightFC_CompositeRank**	37.3	53.51	63.24	68.11	73.51	59.13
**Scaled_WithinStudy_Top1CDG_WeightLog2FC_CompositeRank**	37.3	53.51	63.24	68.11	73.51	59.13
**Scaled_WithinStudy_Top1CDG_Unweighted_CompositeRank**	37.3	53.51	63.24	68.11	73.51	59.13
**Raw_PanStudy_Top1CDG_WeightFC_CompositeRank**	37.84	56.22	62.16	66.49	70.81	58.7
**Raw_PanStudy_Top1CDG_WeightLog2FC_CompositeRank**	37.84	56.22	62.16	66.49	70.81	58.7

**Raw_PanStudy_Top1CDG_Unweighted_CompositeRank**	37.84	56.22	62.16	66.49	70.81	58.7
**Raw_PanStudy_Top1CDG_WeightFC_L2Rank**	37.84	56.22	62.16	66.49	70.27	58.6
**Raw_PanStudy_Top1CDG_WeightLog2FC_L2Rank**	37.84	56.22	62.16	66.49	70.27	58.6
**Raw_PanStudy_Top1CDG_Unweighted_L2Rank**	37.84	56.22	62.16	66.49	70.27	58.6
**Scaled_PanStudy_Top1CDG_WeightFC_CompositeRank**	38.38	52.97	60.54	69.19	70.81	58.38
**Scaled_PanStudy_Top1CDG_WeightLog2FC_CompositeRank**	38.38	52.97	60.54	69.19	70.81	58.38
**Scaled_PanStudy_Top1CDG_Unweighted_CompositeRank**	38.38	52.97	60.54	69.19	70.81	58.38
**Scaled_WithinStudy_Top1CDG_WeightFC_L2Rank**	35.68	51.89	62.16	67.57	72.97	58.05
**Scaled_WithinStudy_Top1CDG_WeightLog2FC_L2Rank**	35.68	51.89	62.16	67.57	72.97	58.05
**Scaled_WithinStudy_Top1CDG_Unweighted_L2Rank**	35.68	51.89	62.16	67.57	72.97	58.05
**Raw_PanStudy_Top5CDGs_Unweighted_L0Rank**	40	50.27	62.16	67.03	69.19	57.73
**Raw_WithinTissue_Top1CDG_WeightFC_CompositeRank**	37.84	56.76	60	64.86	68.65	57.62
**Raw_WithinTissue_Top1CDG_WeightLog2FC_CompositeRank**	37.84	56.76	60	64.86	68.65	57.62
**Raw_WithinTissue_Top1CDG_Unweighted_CompositeRank**	37.84	56.76	60	64.86	68.65	57.62
**Raw_WithinTissue_Top1CDG_WeightFC_L2Rank**	37.84	56.76	60	64.86	67.57	57.41
**Raw_WithinTissue_Top1CDG_WeightLog2FC_L2Rank**	37.84	56.76	60	64.86	67.57	57.41
**Raw_WithinTissue_Top1CDG_Unweighted_L2Rank**	37.84	56.76	60	64.86	67.57	57.41
**Scaled_PanStudy_Top1CDG_WeightFC_L2Rank**	37.3	49.73	58.38	67.03	69.73	56.43
**Scaled_PanStudy_Top1CDG_WeightLog2FC_L2Rank**	37.3	49.73	58.38	67.03	69.73	56.43
**Scaled_PanStudy_Top1CDG_Unweighted_L2Rank**	37.3	49.73	58.38	67.03	69.73	56.43
**Scaled_WithinTissue_Top1CDG_WeightFC_CompositeRank**	33.51	50.81	61.62	67.03	69.19	56.43
**Scaled_WithinTissue_Top1CDG_WeightLog2FC_CompositeRank**	33.51	50.81	61.62	67.03	69.19	56.43
**Scaled_WithinTissue_Top1CDG_Unweighted_CompositeRank**	33.51	50.81	61.62	67.03	69.19	56.43
**Scaled_WithinTissue_Top1CDG_WeightFC_L2Rank**	30.81	48.11	59.46	65.41	68.11	54.38
**Scaled_WithinTissue_Top1CDG_W**	30.81	48.11	59.46	65.41	68.11	54.38

**eightLog2FC_L2Rank**						
**Scaled_WithinTissue_Top1CDG_Unweighted_L2Rank**	30.81	48.11	59.46	65.41	68.11	54.38
**Raw_WithinTissue_Top5CDGs_Unweighted_L0Rank**	34.59	47.03	56.22	61.62	65.41	52.97
**Raw_PanStudy_Top3CDGs_Unweighted_L0Rank**	32.43	41.62	47.03	52.97	56.76	46.16
**Raw_WithinTissue_Top3CDGs_Unweighted_L0Rank**	27.57	35.68	43.24	52.43	57.3	43.24
**Raw_WithinStudy_Top3CDGs_Unweighted_L0Rank**	27.57	36.22	42.7	48.11	56.76	42.27
**Raw_PanStudy_Top1CDG_Unweighted_L0Rank**	10.81	20	25.41	35.14	38.92	26.06
**Raw_WithinStudy_Top1CDG_Unweighted_L0Rank**	9.19	15.14	24.32	31.35	34.59	22.92
**Raw_WithinTissue_Top1CDG_Unweighted_L0Rank**	8.65	14.59	20.54	27.57	31.35	20.54

Supplemental References

1. Muraro, M.J.; Dharmadhikari, G.; Grün, D.; Groen, N.; Dielen, T.; Jansen, E.; van Gurp, L.; Engelse, M.A.; Carlotti, F.; de Koning, E.J.P.; et al. A Single-Cell Transcriptome Atlas of the Human Pancreas. *Cell Syst* **2016**, *3*, 385-394.e3.
2. Grün, D.; Muraro, M.J.; Boisset, J.-C.; Wiebrands, K.; Lyubimova, A.; Dharmadhikari, G.; van den Born, M.; van Es, J.; Jansen, E.; Clevers, H.; et al. De Novo Prediction of Stem Cell Identity using Single-Cell Transcriptome Data. *Cell Stem Cell* **2016**, *19*, 266–277.
3. Segerstolpe, Å.; Palasantza, A.; Eliasson, P.; Andersson, E.-M.; Andréasson, A.-C.; Sun, X.; Picelli, S.; Sabirsh, A.; Clausen, M.; Bjursell, M.K.; et al. Single-Cell Transcriptome Profiling of Human Pancreatic Islets in Health and Type 2 Diabetes. *Cell Metab.* **2016**, *24*, 593–607.
4. Voigt, A.P.; Mulfaul, K.; Mullin, N.K.; Flamme-Wiese, M.J.; Giacalone, J.C.; Stone, E.M.; Tucker, B.A.; Scheetz, T.E.; Mullins, R.F. Single-cell transcriptomics of the human retinal pigment epithelium and choroid in health and macular degeneration. *Proc. Natl. Acad. Sci. U. S. A.* **2019**, *116*, 24100–24107.
5. Immune Cell Atlas: Blood Mononuclear Cells (2 donors, 2 sites) Available online: https://singlecell.broadinstitute.org/single_cell/study/SCP345/ica-blood-mononuclear-cells-2-donors-2-sites (accessed on Sep 1, 2019).
6. Ginhoux, F.; Jung, S. Monocytes and macrophages: developmental pathways and tissue homeostasis. *Nat. Rev. Immunol.* **2014**, *14*, 392–404.
7. Ferdek, P.E.; Jakubowska, M.A. Biology of pancreatic stellate cells-more than just pancreatic cancer. *Pflugers Arch.* **2017**, *469*, 1039–1050.
8. Franzén, O.; Gan, L.-M.; Björkegren, J.L.M. PanglaoDB: a web server for exploration of mouse and human single-cell RNA sequencing data. *Database* **2019**, *2019*, doi:10.1093/database/baz046.
9. Baron, M.; Veres, A.; Wolock, S.L.; Faust, A.L.; Gaujoux, R.; Vetere, A.; Ryu, J.H.; Wagner, B.K.; Shen-Orr, S.S.; Klein, A.M.; et al. A Single-Cell Transcriptomic Map of the Human and Mouse Pancreas Reveals Inter- and Intra-cell Population Structure. *Cell Syst* **2016**, *3*, 346-360.e4.
10. Vieira Braga, F.A.; Kar, G.; Berg, M.; Carpaij, O.A.; Polanski, K.; Simon, L.M.; Brouwer, S.; Gomes, T.; Hesse, L.; Jiang, J.; et al. A cellular census of human lungs identifies novel cell states in health and in asthma. *Nat. Med.* **2019**, *25*, 1153–1163.
11. Deprez, M.; Zaragosi, L.-E.; Truchi, M.; Becavin, C.; Ruiz García, S.; Arguel, M.-J.; Plaisant, M.; Magnone, V.; Lebrigand, K.; Abelanet, S.; et al. A Single-Cell Atlas of the Human Healthy Airways. *Am. J. Respir. Crit. Care Med.* **2020**, *202*, 1636–1645.
12. Wilk, A.J.; Rustagi, A.; Zhao, N.Q.; Roque, J.; Martínez-Colón, G.J.; McKechnie, J.L.; Ivison, G.T.; Ranganath, T.; Vergara, R.; Hollis, T.; et al. A single-cell atlas of the peripheral immune response in patients with severe COVID-19. *Nat. Med.* **2020**, *26*, 1070–1076.
13. Vento-Tormo, R.; Efremova, M.; Botting, R.A.; Turco, M.Y.; Vento-Tormo, M.; Meyer, K.B.; Park, J.-E.; Stephenson, E.; Polański, K.; Goncalves, A.; et al. Single-cell reconstruction of the early maternal-fetal interface in humans. *Nature* **2018**, *563*, 347–353.
14. Aizarani, N.; Saviano, A.; Sagar; Mailly, L.; Durand, S.; Herman, J.S.; Pessaux, P.; Baumert, T.F.; Grün, D. A human liver cell atlas reveals heterogeneity and epithelial progenitors.

Nature **2019**, *572*, 199–204.
15. Ramachandran, P.; Dobie, R.; Wilson-Kanamori, J.R.; Dora, E.F.; Henderson, B.E.P.; Luu, N.T.; Portman, J.R.; Matchett, K.P.; Brice, M.; Marwick, J.A.; et al. Resolving the fibrotic niche of human liver cirrhosis at single-cell level. *Nature* **2019**, *575*, 512–518.
16. Stewart, B.J.; Ferdinand, J.R.; Young, M.D.; Mitchell, T.J.; Loudon, K.W.; Riding, A.M.; Richoz, N.; Frazer, G.L.; Staniforth, J.U.L.; Vieira Braga, F.A.; et al. Spatiotemporal immune zonation of the human kidney. *Science* **2019**, *365*, 1461–1466.
17. Menon, M.; Mohammadi, S.; Davila-Velderrain, J.; Goods, B.A.; Cadwell, T.D.; Xing, Y.; Stemmer-Rachamimov, A.; Shalek, A.K.; Love, J.C.; Kellis, M.; et al. Single-cell transcriptomic atlas of the human retina identifies cell types associated with age-related macular degeneration. *Nat. Commun.* **2019**, *10*, 4902.
18. Han, X.; Zhou, Z.; Fei, L.; Sun, H.; Wang, R.; Chen, Y.; Chen, H.; Wang, J.; Tang, H.; Ge, W.; et al. Construction of a human cell landscape at single-cell level. *Nature* **2020**, *581*, 303–309.
19. Madissoon, E.; Wilbrey-Clark, A.; Miragaia, R.J.; Saeb-Parsy, K.; Mahbubani, K.T.; Georgakopoulos, N.; Harding, P.; Polanski, K.; Huang, N.; Nowicki-Osuch, K.; et al. scRNA-seq assessment of the human lung, spleen, and esophagus tissue stability after cold preservation. *Genome Biol.* **2019**, *21*, 1.
20. Durante, M.A.; Kurtenbach, S.; Sargi, Z.B.; Harbour, J.W.; Choi, R.; Kurtenbach, S.; Goss, G.M.; Matsunami, H.; Goldstein, B.J. Single-cell analysis of olfactory neurogenesis and differentiation in adult humans. *Nat. Neurosci.* **2020**, *23,* 323–326.
21. Seurat - guided clustering tutorial Available online: https://satijalab.org/seurat/articles/pbmc3k_tutorial.html (accessed on Mar 13, 2021).
22. 3k PBMCs from a Healthy Donor (v1), Single Cell Gene Expression Dataset by Cell Ranger 1.1.0, 10x Genomics Available online: https://support.10xgenomics.com/single-cell-gene-expression/datasets/1.1.0/pbmc3k? (accessed on Mar 13, 2021).
